# Developing Clinical Leadership in Orthopedic Nursing: A Qualitative Study to Identify the Components of a Complex Intervention

**DOI:** 10.3390/healthcare14183020

**Published:** 2026-09-15

**Authors:** Flaviu Moldovan, Liviu Moldovan

**Affiliations:** 1 Orthopedics—Traumatology Department, Faculty of Medicine, George Emil Palade University of Medicine, Pharmacy, Science, and Technology of Targu Mures, 540142 Targu Mures, Romania; 2 Faculty of Engineering and Information Technology, George Emil Palade University of Medicine, Pharmacy, Science, and Technology of Targu Mures, 540142 Targu Mures, Romania; liviu.moldovan@umfst.ro

**Keywords:** clinical leadership, orthopedic nursing, orthopedic and trauma clinical nurse specialist, complex intervention, specialist clinical nurse, qualitative research, MRC framework

## Abstract

**Background/Objective:** Clinical leadership is essential for improving care quality and patient safety in complex orthopedic emergency settings. This study examined perceptions of clinical leadership among Orthopedic and Trauma Clinical Nurse Specialists (OTCNSs) at a major regional trauma center in Romania. The objective was to apply and contextually adapt a previously developed methodological approach to identify candidate components for a clinical leadership development intervention tailored to the specific clinical demands of orthopedic trauma care and the Romanian legislative environment. **Methods:** A qualitative approach was utilized, systematically applying and contextually adapting the two-phase methodological architecture developed by Palermo et al. Semi-structured interviews were conducted with seven OTCNSs and analyzed using hybrid deductive–inductive thematic analysis. Following this, a consensus meeting was convened with nine OTCNSs, one Head Nurse, and one Clinical Researcher to co-construct a locally tailored intervention prototype. **Results:** Five overarching themes emerged from the interviews: individual attributes and professional capabilities, interprofessional collaboration and relational dynamics, team frameworks and organizational culture, role-based leadership and functional autonomy, and patient-centered trajectories. Based on these findings and stakeholder consensus, a four-component intervention prototype was co-constructed: (1) clinical orientation pathway and strategic onboarding, (2) core competency curriculum for advanced orthopedic nursing, (3) point-of-care mentorship and reflective clinical support, and (4) collaborative peer circles and case-based reflection. **Conclusions:** This study identified perceived leadership-development needs among OTCNSs and generated a preliminary, contextually tailored intervention prototype. The findings suggest that clinical leadership is shaped by a dynamic interplay of personal, relational, institutional, and patient-centered variables. The candidate intervention components require feasibility testing, refinement, and evaluation before they can be recommended for wider implementation.

## 1. Introduction

Healthcare systems across the globe have undergone a profound transformation in recent decades, driven by ageing populations, the rising burden of chronic diseases, and the imperative to respond to new and complex care needs [1]. These changes have necessitated corresponding developments in nursing, in terms of organisational structures, clinical competencies, and the emergence of new professional roles [2]. Within this evolving landscape, clinical leadership has emerged as a concept of central importance for influencing practice, fostering innovation, and driving improvement in care delivery [3].

Clinical leadership is defined as the process of influencing point-of-care innovation and improvement in organisational processes and care practices to achieve quality and safety of care outcomes [4]. It is distinguished from managerial leadership by the additional requirement of technical competency within the area of practice [5]. Effective clinical leadership has been shown to have a positive impact on the work environment, enhancing nurses’ professional growth and job satisfaction [6]. Clinical skills, effective communication, and the ability to positively influence teams are key attributes of clinical leadership [7]. According to Stanley and Stanley [1], competence acquired through experience and credibility among peers are fundamental attributes of the clinical leader.

Unlike leadership in general, clinical leaders operate at high levels of clinical interaction, without necessarily holding managerial positions [8]. Clinical competence is fundamental to clinical leadership because of its impact at the point of care [9]. Nurses with clinical leadership are effective collaborators and communicators, capable of coordinating patient care. Leadership development should involve all nurses, regardless of their role, through training, mentoring, and organisational support [10]. Promoting a culture of feedback and practical development opportunities can strengthen clinical leadership, improving patient care and the work environment [11].

Clinical leadership is considered crucial for addressing issues such as complexity, change, safety and quality of care, and is essential for a positive work environment and professional development [12]. It can be considered a shared responsibility, exercised by every healthcare professional regardless of their position in the healthcare system [13]. Leadership training can improve the hospital work environment by promoting team competence and decision-making autonomy [14]. Although nurses believe that formal qualifications are necessary to develop leadership skills, leadership activities at the patient’s bedside are an integral part of nursing practice [15]. Nurses with greater clinical experience are often considered informal leaders, able to influence their colleagues even without holding a formal role [16].

Orthopedic nursing presents a distinctive set of challenges that render clinical leadership particularly vital. Orthopedic departments within emergency hospitals manage a high volume of patients with complex and often urgent conditions, ranging from fragility fractures in older adults to polytrauma in younger patients following road traffic accidents or falls from height [17]. These patients require multifaceted care pathways that encompass acute pain management, surgical intervention, rehabilitation, and prevention of complications such as venous thromboembolism, pressure injuries, and infections [18,19].

The orthopedic setting is inherently interdisciplinary, requiring close collaboration among orthopedic surgeons, anesthesiologists, physiotherapists, occupational therapists, social workers, and nursing staff [20]. Effective coordination of this multidisciplinary team is essential for ensuring safe, efficient, and timely treatment [21]. Clinical leadership in this context involves not only advanced clinical knowledge and technical proficiency but also the ability to coordinate complex patient care situations, facilitate communication across professional boundaries, and drive evidence-based practice implementation [22].

Moreover, orthopedic nurses often work in high-pressure environments with significant patient throughput, unpredictable admissions, and the constant need to balance competing priorities [23]. In such settings, clinical leaders, whether in formal or informal roles, play a crucial part in modelling evidence-based, person-centered practice, coaching colleagues, driving quality and safety initiatives, and motivating teams through periods of change [24]. The development of robust clinical leadership capacity within orthopedic nursing is therefore not merely an aspirational goal but an operational necessity for maintaining safe, high-quality patient care [25].

The Targu Mures County Emergency Clinical Hospital serves as a major regional trauma and orthopedic center in the Transylvania region of Romania [26]. Its Orthopedics and Traumatology Clinic manages a high volume of emergency and elective orthopedic cases, functioning as a tertiary referral center for the surrounding region.

Romania has made notable strides in recent years to advance nursing leadership at a national level. In 2022, the country implemented its first national nursing leadership programme, designed to move leadership development beyond individual training toward broader organizational change. This initiative reflects a growing recognition within Romanian healthcare policy of the strategic importance of nursing leadership for building resilient health systems [27]. European healthcare systems are placing increasing emphasis on advanced competencies and the independent practice of nurses, principles that have already been incorporated into Romanian legislation [28]. The Romanian Order of General Nurses, Midwives and Medical Assistants (OAMGMAMR) has been actively engaged in promoting professional autonomy, leadership, and innovation in healthcare [29].

Despite these national-level developments, a significant gap persists at the clinical unit level. Within the Orthopedics and Traumatology Department of the Targu Mures County Emergency Clinical Hospital, there is currently no structured, evidence-based pathway specifically designed to foster clinical leadership among Orthopedic and Trauma Clinical Nurse Specialists (OTCNSs). While these professionals are expected to demonstrate clinical leadership through activities including coordination of complex patient care situations, intra- and interprofessional collaboration, and facilitation of change within the team, no formal programme exists to systematically develop these competencies. This mirrors a broader challenge identified in the literature: despite the priority given to leadership development programmes, there is limited evidence regarding the specific strategies that could be implemented to promote the development of clinical leadership among healthcare professionals [30].

It is widely suggested that nurse leaders should reflect on and take responsibility for the development of their own leadership expertise [31]. Involving OTCNSs in this process is therefore both appropriate and essential. As highlighted by a systematic review on nurses’ clinical leadership in hospital settings, interventions designed to promote clinical leadership are inherently complex, requiring that cognitive, interpersonal, and intrinsic competencies, as well as psychological empowerment, emotional intelligence, and critical reflexivity skills, be addressed [32]. The development of multicomponent, theory-based and mixed-format programmes is recommended [33].

Given this knowledge gap and the absence of a structured leadership development pathway within the local context, this study was designed with the following specific objectives:

1. To explore how Orthopedic and Trauma Clinical Nurse Specialists (OTCNSs) within orthopedics and traumatology perceive their professional roles and clinical leadership domains.
2. To pinpoint the specific contextual factors and behaviors that support the growth of clinical leadership within this orthopedic environment.
3. To examine the extent to which clinical leadership development initiatives are currently integrated into the routine daily practice of these OTCNSs.
4. To outline the core elements required for a complex leadership-promoting intervention, structured around the Medical Research Council (MRC) framework guidelines.

The purpose of this study is therefore to examine OTCNSs’ perceptions of their role and clinical leadership within the orthopedic emergency context, and to identify the components of a complex, co-constructed intervention aimed at improving clinical leadership. By grounding the intervention in the lived experiences and articulated needs of the professionals themselves, and by aligning it with both international evidence and national policy directions, this research seeks to contribute a contextually relevant and sustainable pathway for clinical leadership development in orthopedic centers.

## 2. Materials and Methods

### 2.1. Study Design

The study was executed using a generic qualitative design over a six-month period between February 2026 and July 2026. This study was prospectively designed as a contextual adaptation and empirical application of the methodological approach developed by Palermo et al. [34], rather than as a fully independent or de novo qualitative inquiry. The decision to adopt this framework was made prior to data collection, based on the following considerations: (1) the original study provided a robust, systematically developed two-phase methodology for identifying intervention components for clinical leadership development among specialist clinical nurses; (2) the framework’s emphasis on stakeholder involvement and contextual adaptation made it suitable for application in a different healthcare system and clinical specialty; and (3) applying the framework in a new context would allow for empirical testing of its transferability while generating context-specific knowledge for the Romanian orthopedic setting.

The present study should therefore be understood as a contextual adaptation that systematically applied the Palermo et al. [34] methodological architecture to a distinctly different clinical and cultural environment, a high-volume tertiary trauma center in Romania, as opposed to the multi-specialty setting of the original study. This adaptation was designed to generate new empirical knowledge about clinical leadership in orthopedic trauma nursing specifically, and to develop an intervention prototype tailored to the Romanian legislative and organizational context.

By bypassing the strict conceptual requirements of traditional qualitative frameworks, such as grounded theory or ethnography, this non-categorical inquiry ensures an expansive and nuanced description of the phenomenon under investigation [35]. This approach is particularly suitable when the research seeks to understand participants’ experiences and perceptions directly, and when the findings are intended to inform practical intervention development in a specific context [36].

To guide the development of complex interventions, the study follows the Medical Research Council framework for developing and evaluating complex interventions. The MRC framework, most recently updated in 2021 through a joint initiative of the UK Medical Research Council and the National Institute for Health Research [37], structures complex intervention research through four interrelated and overlapping phases: intervention development or identification, feasibility, evaluation, and implementation. Each phase incorporates key elements that must be considered continuously, including context, relevant theory, stakeholder involvement, identification of uncertainties, refinement of the intervention, and economic considerations. These elements must be integrated and revised iteratively to effectively guide the process of creating the complex intervention.

The rationale for selecting the MRC framework is that clinical leadership development is inherently a complex, multifactorial phenomenon involving multiple interacting variables, including personal characteristics, interpersonal dynamics, organizational culture, and role-specific competencies. Complex interventions are those built up from several components that may act both independently and interdependently [38]. The framework’s emphasis on stakeholder involvement and contextual adaptation makes it particularly appropriate for co-constructing an intervention with the very professionals who will ultimately implement it.

An expert consensus meeting was conducted to establish the fundamental components necessary for co-designing the complex intervention [39]. Consensus methods are increasingly used in healthcare research to synthesize expert opinions and develop statements, guidelines, or intervention components that reflect the collective judgement of stakeholders [40].

#### 2.1.1. Relationship to the Palermo et al. Framework

The methodological design of this study was prospectively based on the two-phase approach described by Palermo et al. [34]. The following elements were predetermined from the source framework and adapted to the Romanian orthopedic context.

The interview schedule was translated and culturally adapted from the semi-structured interview guide published in the original study. The seven key dimensions: understanding of clinical leadership, identification of clinical leaders, factors fostering/hindering leadership, personal reflection, interprofessional collaboration, impact on patient care, and personal satisfaction, were retained as the structural backbone, but questions were rephrased to reflect the specific terminology and practice realities of Romanian orthopedic nursing, for example references to national legislation [28], the specific role designation of OTCNS, and the organizational structure of the Targu Mures trauma center.

The two-phase approach, comprising individual qualitative interviews followed by a multi-stakeholder consensus meeting, was adopted from Palermo et al. as the overarching methodological structure. However, the specific implementation was culturally and structurally customized to reflect the professional competencies mandated by Romanian Law no. 278/2015 [28] and the clinical peculiarities of tertiary trauma care.

The following elements emerged independently from the Romanian data and were not predetermined by the source framework:

(a) thematic content: While the macro-structure of five overarching themes corresponds to the original study, the specific sub-themes, their content, and their relative emphasis reflect the distinctive experiences of OTCNSs in a Romanian trauma center. For example, sub-themes such as “Statutory mandates and structural accountability” and “The orthopedic clinical ecosystem and workplace climate” emerged from participants’ discussions of the Romanian legislative context and the specific pressures of high-volume trauma care, which were not present in the original study.
(b) intervention details: The specific operational elements of the intervention, including the six-month mentorship period, one hour of protected time per week, monthly interdisciplinary case forums, and quarterly reflective practice sessions, were derived from consensus discussions with Romanian stakeholders, not from the source framework. Similarly, the four curriculum modules: Foundations of Clinical Leadership, Effective Communication and Interprofessional Collaboration, Evidence-Based Orthopedic Practice, and Empowerment and Change Management, were tailored to Romanian continuing education structures and the specific clinical protocols of the department.
(c) legislative and policy integration: The explicit integration of Romanian Law no. 278/2015, Government Emergency Ordinance no. 144/2008, and references to the Order of General Nurses, Midwives and Medical Assistants of Romania reflect a contextual adaptation that was not present in the original study.

The formal adaptation process followed a structured approach. First, the interview guide was translated and reviewed by a bilingual researcher with expertise in qualitative methods. Second, the questions were piloted with one OTCNS to assess cultural and linguistic appropriateness, leading to minor refinements in wording. Third, the coding framework was developed through a hybrid deductive–inductive approach, combining predetermined domains from the adapted interview framework with emergent codes from the Romanian data. Fourth, the consensus meeting structure was adapted to include reference to Romanian legislation and professional regulations, and to ensure representation of the specific clinical sub-specialties within the orthopedic trauma department.

It is important to note that the original Palermo et al. study was a single qualitative development study, not a validated framework in the psychometric or empirically established sense. The present study therefore does not claim to validate this framework, but rather to apply and adapt a promising methodological approach to generate contextually relevant knowledge and a preliminary intervention prototype.

The Palermo et al. study was accessed as an accepted manuscript/pre-publication version during the development of this study’s protocol in late 2025. The authors obtained permission from the corresponding author of Palermo et al. to adapt the interview guide, methodological approach, and conceptual framework for the Romanian context. The final published version [34] was subsequently cited upon its release in May 2026.

#### 2.1.2. Researcher Characteristics and Reflexivity

All semi-structured interviews were conducted by the first author, a male researcher with a PhD in Medicine and MSc in Quality Management systems from the George Emil Palade University of Medicine, Pharmacy, Science, and Technology of Targu Mures. At the time of the study, the first author was employed as a university lecturer at the Faculty of Medicine and held an affiliate position within the Orthopedics and Traumatology Department, where he had been involved in clinical research collaboration for approximately three years prior to this study. The first author had completed formal training in qualitative research methods, including a university-certified course in qualitative data analysis and thematic analysis with a duration of 40 h, and had previously conducted and published qualitative research in healthcare settings.

The second author, a male researcher with a PhD in Engineering and Professor at the Faculty of Engineering and Information Technology, served as the senior supervisor for the study. He provided methodological oversight, participated in the coding review sessions, and contributed to the interpretation of findings. Neither author had clinical nursing qualifications or direct clinical responsibilities in the department.

At the time of study commencement, the first author had established professional relationships with some members of the Orthopedics and Traumatology Department through prior collaborative research projects. These relationships were professional rather than personal, and the first author held no hierarchical authority over the OTCNS participants, as he was not a member of the nursing or clinical management structure. However, participants were aware of the first author’s affiliation with the department and his role as a researcher. To minimize potential power imbalances and social desirability bias, the following measures were implemented:

- Participants were informed that their participation was entirely voluntary and that declining or withdrawing would have no consequences for their professional standing or working relationships.
- Interviews were conducted in a private setting outside the clinical work area, with no nursing managers or departmental leaders present.
- Participants were assured of confidentiality and that their individual responses would not be shared with department leadership.
- The interview guide was designed with open-ended, non-judgmental questions to minimize leading or value-laden prompts.
- The consensus meeting was facilitated with explicit attention to ensuring all participants had opportunities to contribute, and the Head Nurse was asked to allow junior staff to speak first on each agenda item to reduce hierarchical influence.

The research team held the following assumptions and interests prior to data collection: (1) that clinical leadership is a valuable and potentially underdeveloped dimension of orthopedic nursing practice; (2) that the Romanian legislative framework provides a foundation for enhanced nursing autonomy that may not be fully realized in practice; and (3) that a structured intervention could support clinical leadership development. These assumptions were discussed explicitly among the research team prior to data collection and were periodically revisited during analysis to recognize their potential influence on interpretation. Regular reflexivity meetings were held between the first and second authors to document and critically examine how these assumptions might shape coding and theme development.

The first author’s insider position, as a researcher with prior collaborative relationships in the department, offered both advantages and potential challenges. Advantages included established trust, familiarity with the clinical context, and the ability to interpret participant accounts with contextual understanding. Potential challenges included the risk that participants might provide socially desirable responses or assume that the research had institutional endorsement. To mitigate these risks, the research team emphasized the independent, academic nature of the study; clearly distinguished the research role from any clinical or administrative role; and used member-checking to verify that interpretations accurately reflected participant perspectives.

### 2.2. Study Site and Participant Profile

The study is conducted in the Orthopedics and Traumatology Department of the Targu Mures Emergency County Clinical Hospital, a major public multi-specialty tertiary referral and trauma center in the Transylvania region of Romania. The department manages a high volume of both emergency and elective orthopedic cases, functioning as a referral center for the surrounding region.

The participants involved in the study comprise Orthopedic and Trauma Clinical Nurse Specialists working within the Orthopedics and Traumatology Department. These professionals perform both direct and indirect patient care activities. Direct care includes comprehensive nursing assessment, perioperative and postoperative management, pain management, prevention of complications, such as venous thromboembolism, pressure injuries, and infections, and patient education regarding mobility, rehabilitation, and discharge planning. Indirect care activities include supporting the broader nursing team through clinical guidance and mentorship, contributing to the implementation of evidence-based guidelines and protocols, collaborating with nurse managers and the wider hospital organization, and facilitating quality improvement initiatives. The proportion of time dedicated to indirect activities typically varies between 20% and 60% of total monthly working hours.

#### Definition and Legal Status of the Orthopedic and Trauma Clinical Nurse Specialist

The Orthopedic and Trauma Clinical Nurse Specialist (OTCNS) is a specialized nursing role within the Romanian healthcare system, responsible for providing advanced nursing care to patients with musculoskeletal conditions, trauma injuries, and related comorbidities. The role is situated within the broader framework of specialist nursing practice recognized under Romanian law.

The legal basis for nursing practice in Romania is established by Government Emergency Ordinance no. 144/2008 regarding the exercise of the profession of general practitioner nurse, the profession of midwife, and the profession of nurse, as well as the organization and functioning of the Order of General Nurses, Midwives and Medical Assistants in Romania, approved with modifications by Law no. 53/2014, and amended by Law no. 278/2015 [28].

The activities exercised under the professional title of nurse include: determining general care needs and providing general, preventive, curative and rehabilitation care services, based on the acquired competence in independently determine the need for healthcare, planification, organization and performing of these service; first aid based on the competence in independently initiating immediate life-saving actions and applying these measures in crisis or calamity situations; reporting specific undertaken activities and independent analysis of the quality of the healthcare provided, in order to improve the professional practice of nurses.

The OTCNS designation in this study refers to a registered nurse who meets the following criteria: (1) completion of post-secondary or university-level nursing education as a basic nursing qualification; (2) recognition as a specialist nurse by the Order of General Nurses, Midwives and Medical Assistants in Romania (OAMGMAMR), obtained through one or more of the following pathways: (a) postgraduate specialist courses in orthopedics and traumatology nursing; (b) successful completion of the professional competency examination in the relevant specialty; (c) a Diploma of Advanced Studies (DAS) or Master’s degree in nursing with an orthopedic specialization; or (d) accumulated clinical experience combined with specialist training as recognized by OAMGMAMR; (3) active membership in OAMGMAMR as professional registration; and (4) current employment in the specialist orthopedic and trauma nursing role within the department for at least one year. It is important to distinguish between the functional OTCNS role designation used in this study and the credentialed specialist nurse status recognized by OAMGMAMR. The OTCNS designation in this study is operationally defined by the nurse’s employment in the specialist role within the Orthopedics and Traumatology Department, encompassing both direct and indirect care responsibilities as described above. Specialist nurse status, by contrast, is a formal credential that may be obtained through multiple pathways, not exclusively through DAS/Master’s qualifications.

The OTCNS scope of practice encompasses both direct and indirect patient care activities. Direct care consists of comprehensive nursing assessment, perioperative and postoperative management, pain management, prevention of complications, and patient education regarding mobility, rehabilitation, and discharge planning. Indirect care consists of supporting the broader nursing team through clinical guidance and mentorship, contributing to the implementation of evidence-based guidelines and protocols, collaborating with nurse managers and the wider hospital organization, and facilitating quality improvement initiatives.

The OTCNS role is distinguished from that of a general nurse working in orthopedics across several dimensions. General nurses typically hold a basic nursing qualification with variable clinical experience, provide direct patient care under supervision, have limited leadership responsibilities, and practice under the direction of senior staff. In contrast, OTCNSs hold additional postgraduate specialist qualifications, have a minimum of one year, typically 3–10 years of specialized orthopedic experience, perform both direct and indirect care with independent clinical decision-making in defined areas, provide clinical guidance and mentorship, drive quality improvement initiatives, and dedicate 20–60% of their time to indirect care activities. This distinction is supported by the legal framework of OUG 144/2008, which recognizes different levels of nursing practice based on acquired competence [28].

### 2.3. Participant Selection and Recruitment Strategy

In establishing the sampling frame and eligibility, we started from the situation of the Orthopedics and Traumatology Department, which employs a total of 14 Orthopedic and Trauma Clinical Nurse Specialists (OTCNSs) at the time of the study. All 14 OTCNSs were assessed for eligibility based on the following inclusion criteria: (1) current employment as an OTCNS in the department for at least one year; (2) willingness to participate and provide informed consent; and (3) ability to communicate effectively in Romanian. Exclusion criteria included OTCNSs on extended leave during the data collection period. All 14 OTCNSs met the inclusion criteria.

Eligibility for participation in this study was based on the functional OTCNS role designation, current employment in the specialist orthopedic and trauma nursing role for at least one year, rather than on the specific credentialing pathway through which specialist nurse status had been obtained. This reflects the study’s focus on the lived experience of nurses occupying the specialist role in the department, rather than on the credentialing route by which they achieved that role. Participants held a range of qualifications, including post-secondary nursing education with specialist certification, DAS/Master’s degrees, and other forms of postgraduate specialist training recognized by OAMGMAMR.

When sampling in the interview phase, for the semi-structured interviews, purposeful sampling according to reasoned choice was adopted. This strategy involved deliberately selecting individuals considered rich information sources on the phenomenon of interest, with attention to diversity in clinical sub-specialties like trauma, elective joint replacement, outpatient fracture clinics, and experience levels, and gender. Seven OTCNSs were purposively selected from the 14 eligible OTCNSs to achieve this diversity while maintaining a manageable sample for in-depth qualitative analysis. All seven invited OTCNSs agreed to participate and provided informed consent. No refusals occurred. The sample size was guided by the principle of data saturation within this specific departmental context, defined as the point at which no new relevant information emerges from additional interviews within this narrowly defined sample. Saturation was assessed concurrently with data collection through iterative review of transcripts and coding.

When sampling within consensus meetings, the sample was purposively constructed to include a broader and more heterogeneous group of stakeholders, reflecting multiple perspectives on clinical leadership, including clinical, managerial, and research. The sample included:

- Nine OTCNSs from the Orthopedics and Traumatology Department, representing diverse clinical sub-specialties and levels of experience. Of these nine, the seven interviewees were included, plus two additional OTCNSs who had not participated in the interviews to ensure broader representation of departmental perspectives.
- One Head Nurse from the Orthopedics Department, providing the managerial perspective and insight into organizational structures.
- One Clinical Researcher from the George Emil Palade University of Medicine, Pharmacy, Science, and Technology of Targu Mures with expertise in nursing leadership and implementation science, providing the research perspective.

All 11 invited consensus meeting participants agreed to participate. No refusals occurred. Participants were contacted individually by the first author, who also organized and facilitated the consensus meeting.

It is important to note that there was some overlap in participants between phases. The seven OTCNS who participated in the interviews represented a subset of the nine OTCNS who participated in the consensus meeting. The two additional OTCNSs in the consensus meeting were purposefully included to broaden the range of perspectives and ensure that the intervention design benefited from input beyond the initial interviewees. The pilot interview participant, one OTCNS not included in the final sample, did not attend the consensus meeting.

### 2.4. Primary Data Generation: Interviews and Consensus Meetings

Semi-structured interviews were conducted between February 2026 and April 2026. Interviews took place in a private office within the Orthopedics and Traumatology Department, away from the clinical work area, to ensure privacy and minimize interruptions. Each interview was conducted face-to-face by the first author. No one other than the participant and the interviewer was present during any interview. Prior to each interview, participants were provided with an information sheet explaining the study purpose, the voluntary nature of participation, confidentiality protections, and their right to withdraw at any time [41,42]. Written informed consent was obtained from all participants.

To explore OTCNSs’ perceptions of their role and clinical leadership, we developed a semi-structured interview schedule adapted from the work of Palermo et al. [34]. The interview schedule comprised 19 open-ended questions organized across seven key dimensions: (1) understanding of clinical leadership (2 items), (2) identification of clinical leaders in orthopedics (3 items), (3) factors fostering and hindering clinical leadership (3 items), (4) personal reflection on role and clinical leadership (3 items), (5) interprofessional collaboration and team dynamics (3 items), (6) impact on patient care (2 items), and (7) personal satisfaction and professional development (3 items). The full interview guide is provided in Appendix A.

The interview guide was pilot-tested with one OTCNS who met the inclusion criteria but was not included in the final sample. The pilot interview served to assess the clarity, cultural appropriateness, and comprehensiveness of the questions, and to refine the wording of several items. Minor adjustments were made to the phrasing of questions to improve clarity and to incorporate Romanian professional terminology. The pilot interview was not included in the analysis.

Each interview began with a brief introduction reiterating the study purpose and confirming the participant’s consent to audio-record the session. Interviews were audio-recorded using a digital recorder with the participant’s permission. The interviewer used open-ended questions and probes to encourage detailed responses, while remaining attentive to participants’ verbal and non-verbal cues. Field notes were made immediately after each interview, documenting the interviewer’s initial impressions, the setting, any contextual factors that may have influenced the interaction, and methodological observations. No repeat interviews were conducted, as data saturation was achieved with the initial set of seven interviews. The duration of interviews ranged from 28 to 55 min (mean: 40 min).

All interviews were conducted in Romanian, audio-recorded, and transcribed verbatim in Romanian by the first author. Transcripts were checked for accuracy against the audio recordings by the first author, with a random sample of 20% of transcripts reviewed by the second author to verify accuracy. For analysis, transcripts were analyzed in Romanian to preserve linguistic and conceptual nuances. Quotations selected for inclusion in the manuscript were translated into English by the first author, a bilingual researcher with native proficiency in Romanian and advanced proficiency in English. To ensure translation accuracy and conceptual equivalence, a second bilingual researcher independently back-translated a selection of quotations into Romanian, and any discrepancies were discussed and resolved through consensus.

Interviews were conducted and transcribed sequentially, with preliminary analysis commencing after the first interview. The research team reviewed transcripts iteratively to assess the emergence of new codes and themes. Saturation was prospectively defined as the point at which three consecutive interviews yielded no new code or thematic content relevant to the research objectives. Saturation was assessed by the first author after each transcription and confirmed through discussion with the senior author. Saturation was achieved after the sixth interview; one additional interview was conducted to confirm that no new information emerged, which was confirmed. No further interviews were conducted after saturation was confirmed.

Preliminary findings, including the initial thematic framework and a summary of key codes and sub-themes, were presented to a subgroup of three interview participants for member checking. Participants were asked to comment on the accuracy and resonance of the interpretations, and to identify any misinterpretations or missing elements. All three confirmed that the themes accurately reflected their experiences and perspectives. No substantive corrections were requested. Full transcripts were not returned to participants to maintain the focus on thematic interpretation rather than individual-level verification.

Following the thematic analysis, a three-hour consensus meeting was convened in May 2026, structured according to the principles of consensus development methodology [39,40] and reported in accordance with the ACCORD guideline for reporting consensus methods [43]. The meeting was held in a conference room at the George Emil Palade University of Medicine, Pharmacy, Science, and Technology of Targu Mures. The meeting was facilitated by the first author, with the second author present as an observer and to provide methodological support. The meeting commenced with a presentation of the interview findings and a brief review of relevant literature. Participants were then invited to discuss the findings, identify gaps, and co-construct intervention components. The discussion was audio-recorded and transcribed, and field notes were maintained by the first author. No one other than the 11 consensus participants and the two authors were present.

#### 2.4.1. Saturation Assessment

Data saturation was systematically assessed throughout the interview phase, with the understanding that saturation claims in qualitative research are necessarily contextual and bounded by the specific sample and setting. To this end, we implemented a procedure in which we defined saturation as the point at which three consecutive interviews did not generate new codes, sub-themes, or thematic content relevant to the research objectives within this narrowly defined departmental sample [35,44]. In the next stage of concurrent analysis, the interviews were transcribed and analyzed sequentially, with the first author performing preliminary coding within 48 h of each interview. We continued with iterative review, in which after each set of three interviews, the research team met to review the codebook, assess whether new codes emerged, and assess the complexity of the thematic structure. Saturation was assessed after the fourth interview as a preliminary indication, after the fifth for ongoing monitoring, and definitively after the sixth when no new codes emerged. An additional interview was conducted to confirm saturation within this sample, which was achieved. For documentation, we kept a saturation log, in which we recorded the codes present after each interview and the point at which no new codes emerged. Because saturation was achieved and confirmed with the seventh interview within this specific departmental context, no further interviews were planned or conducted. It is important to note that this saturation claim applies to the narrowly defined sample of OTCNSs in this single department and does not imply broader thematic exhaustiveness regarding clinical leadership in orthopedic nursing more generally.

#### 2.4.2. Consensus Meeting Procedure

The consensus meeting was designed as a structured stakeholder consultation within this local group, informed by established consensus development methodologies. While not a formal Delphi or nominal group technique, the meeting incorporated several elements of structured consensus processes to ensure systematic and transparent decision-making. Four weeks prior to the meeting, all participants received a pre-meeting package containing: a meeting agenda (provided in Supplementary Material Agenda S1); a summary of the interview findings, including the thematic framework and key quotations; a list of candidate intervention components derived from the interview findings, organized by theme; a brief literature review on clinical leadership development interventions; and relevant excerpts from Romanian legislation Law no. 278/2015 and Government Emergency Ordinance no. 144/2008.

Consensus was prospectively defined as agreement by at least 80% of participants on the inclusion and specification of each intervention component. The following decision rules were established in advance: each proposed component would be discussed, and participants would indicate their agreement through a show of hands; components with ≥80% agreement would be included in the intervention; components with 50–79% agreement would be discussed further and refined before a second vote; components with <50% agreement would be excluded or substantially revised; any participant could request anonymous voting if they felt uncomfortable with public voting. Prior to the meeting, participants were asked to disclose any conflicts of interest relevant to the intervention development. No conflicts of interest were declared by any participant.

The meeting was facilitated by the first author, with the second author present as an observer. To minimize the potential for senior or managerial participants to dominate the discussion, the following measures were implemented: Participants were informed at the outset that all perspectives were equally valued and that the goal was to reach a collective consensus; The Head Nurse was asked to allow junior participants and OTCNSs to speak first on each agenda item; Small-group brainstorming was conducted in mixed groups to ensure diverse perspectives were represented; The facilitator actively encouraged contributions from quieter participants and periodically summarized the range of views expressed; When divergent views emerged, the facilitator invited additional perspectives and ensured that differing opinions were documented; Anonymous voting was offered as an option, although all participants chose public voting.

The consensus meeting was audio-recorded, and the first author maintained detailed field notes documenting the discussion, the range of views expressed, any disagreements, and the rationale for decisions. These field notes were reviewed by the second author to verify the accuracy of the documentation.

#### 2.4.3. Literature Review for Consensus Meeting Preparation

A targeted literature review was conducted to inform the consensus meeting discussions and to ensure that the proposed intervention components were grounded in existing evidence. The review was designed as a targeted/contextual review rather than a full systematic review, given the specific purpose of identifying evidence-based elements for clinical leadership development interventions that could be adapted to the Romanian orthopedic context.

The review aimed to identify evidence-based components of effective clinical leadership development interventions, best practices in nursing leadership education and training, implementation strategies for leadership development in clinical settings, and relevant frameworks for evaluating leadership interventions.

The following databases were searched: PubMed/MEDLINE, CINAHL (Cumulative Index to Nursing and Allied Health Literature), Scopus, and Web of Science.

Additionally, reference lists of identified articles were hand-searched to identify additional relevant studies. Grey literature was not systematically searched, although key organizational reports, for example from the World Health Organization or the European Federation of Nurses Associations, were consulted where relevant.

The search covered publications from January 2010 to December 2025, reflecting the timeframe in which most of the evidence on clinical leadership development interventions has emerged, while also allowing for the inclusion of foundational studies published prior to 2010.

The search strategy combined terms related to clinical leadership (for example, clinical leadership, nursing leadership, leadership development), intervention design (for example, complex intervention, leadership intervention, training program), setting (for example, hospital, clinical setting, orthopedic nursing), and outcomes (for example, leadership competencies, patient outcomes, organizational outcomes).

Inclusion criteria consist in peer-reviewed articles published in English; studies reporting on leadership development interventions for nurses in clinical settings; empirical studies including quantitative, qualitative, or mixed-methods; systematic reviews and meta-analyses; studies published from 2010 onwards. Exclusion criteria were opinion pieces, editorials, and conference abstracts; studies focused exclusively on managerial leadership rather than clinical leadership; studies conducted in non-clinical settings; articles not available in English.

In the study selection process, titles and abstracts were screened by the first author for relevance to the research objectives. Full-text articles were retrieved for potentially relevant studies and assessed against the eligibility criteria. The second author independently reviewed a random sample of 20% of the screened articles to verify the consistency of the selection process. Discrepancies were resolved through discussion.

Findings from the included studies were synthesized narratively, with key themes organized around core components of effective leadership interventions (for example, mentorship, training, peer support), implementation considerations (for example, protected time, organizational support), and evaluation approaches (for example, outcome measures, feasibility testing).

The literature review informed the development of the interview schedule by identifying key dimensions of clinical leadership that should be explored, such as understanding of clinical leadership, interprofessional collaboration, impact on patient care, personal satisfaction, and professional development. This ensured that the interview guide was grounded in existing evidence while remaining open to context-specific findings.

The literature review findings were presented at the consensus meeting, as a 10 min overview, to provide participants with an evidence-based foundation for their discussions. This presentation ensured that participants were aware of what has been shown to be effective in other settings, which informed their deliberations about which components would be appropriate for the Romanian orthopedic context.

The literature review directly informed the structure and content of the intervention components. The four-component structure including orientation, curriculum, mentorship, and peer circles, was informed by evidence from the literature on comprehensive leadership development approaches [32,41,42]. The specific curriculum modules were informed by evidence on the competencies required for clinical leadership, including communication, evidence-based practice, and change management [3,7,22,23]. The mentorship component was informed by evidence on the effectiveness of mentorship and coaching in leadership development [32]. The peer learning component was informed by evidence on communities of practice and reflective learning [6].

### 2.5. Data Synthesis and Analytical Framework

The transcribed interviews were analyzed using a six-phase thematic analysis framework, drawing from the methodological guidance of Vaismoradi et al. [44]. This method was selected for its flexibility and capacity to identify, analyze, and report patterns within qualitative data. The analysis proceeded through the following phases: (1) familiarization with the data through repeated reading of transcripts; (2) generation of initial codes; (3) searching for themes by collating codes; (4) reviewing themes; (5) defining and naming themes; and (6) producing the report.

The hybrid approach was deliberately chosen to balance the need for systematic exploration of predetermined domains (derived from the Palermo et al. framework) with openness to emergent, context-specific findings from the Romanian data. The deductive elements provided an initial organizational structure for the data, while the inductive elements ensured that the analysis remained grounded in participants’ experiences and allowed for the identification of themes not captured by the source framework. The deductive dimension was informed by the research objectives and the adapted interview framework, which provided broad thematic domains—for example, understanding of clinical leadership, interprofessional collaboration, and impact on patient care—that guided initial data organization. The inductive dimension allowed for the emergence of specific codes, sub-themes, and content from the data that were not predetermined by the source framework. This hybrid approach was appropriate given the study’s design as a contextual adaptation of an existing methodological framework [34], while remaining open to context-specific findings.

The coding process followed a structured procedure. The first author conducted the initial coding of all seven transcripts. This involved line-by-line reading and assignment of codes to meaningful segments of text. Codes were generated through a hybrid deductive–inductive approach: deductive codes were derived from the predetermined domains of the adapted interview framework, while inductive codes emerged from the language and meaning of participant accounts. ATLAS.ti 23 software [45] was used to manage the coding process.

To enhance analytical rigor, the second author independently reviewed a random sample of three transcripts, approximately 40% of the total data, using the preliminary codebook developed by the first author. This independent review served two purposes: (1) to assess the consistency and comprehensiveness of the coding; and (2) to identify any additional codes or alternative interpretations not captured in the initial coding.

Coding reconciliation followed the independent review. The two authors convened to compare coding decisions, discuss discrepancies, and refine the codebook. Discrepancies were resolved through discussion until consensus was reached. In instances where disagreement persisted, the code was discussed with a third researcher not involved in the study, which is a qualitative methods expert, to provide an independent perspective. No major disagreements occurred that could not be resolved through discussion.

Following codebook refinement, the first author grouped codes into potential sub-themes based on patterns, relationships, and recurring concepts. The preliminary thematic structure was then reviewed and refined through discussion between both authors. The second author provided methodological oversight and challenged the first author’s interpretations to ensure they remained grounded in the data rather than reflecting preconceptions. The final themes were defined and named through collaborative discussion.

In an independent multi-coder review session, as part of the quality assurance process, the research team conducted two “multi-coder review sessions” where the first and second authors together reviewed the coding and thematic structure across all transcripts. These sessions involved: (a) reviewing the application of codes to specific quotations; (b) examining the coherence and distinctiveness of sub-themes; and (c) assessing whether the overarching themes adequately captured the range of participant experiences. These sessions were not independent coding of all transcripts, but rather collaborative review and refinement of the analytical framework.

It is important to distinguish between the initial coding phase which was conducted independently by the first author and the multi-coder review sessions which was conducted collaboratively by both authors. The initial coding established the foundational codebook, while the review sessions served to review, refine, and challenge the coding decisions through collaborative discussion. This two-stage approach ensured both consistency in initial coding and rigor through independent verification of a subset of transcripts.

To illustrate the coding process, an example is provided in Table S2 of the Supplementary Material, showing how raw data extracts progressed through initial codes to sub-themes and overarching themes. The analysis generated 73 initial codes, which were subsequently grouped into 19 sub-themes. These sub-themes were then organized into 5 overarching themes that captured the core dimensions of clinical leadership as perceived by OTCNSs.

#### Analysis of Consensus Meeting Data

The consensus meeting was audio-recorded and transcribed verbatim. The transcript was analyzed using content analysis to identify key discussion points, areas of agreement, and proposed intervention components. The first author reviewed the transcript to extract all proposed intervention elements and the rationale provided by participants. The second author independently reviewed the transcript to verify the completeness and accuracy of the extracted elements. Any discrepancies were resolved through discussion. The final intervention components were developed by integrating: (a) the thematic analysis findings from the interviews; (b) the consensus meeting discussion; (c) relevant literature; and (d) Romanian legislative requirements.

Each final intervention component was linked to its source(s) through a systematic process:

- Interview-derived components: Components that emerged from the thematic analysis were identified by mapping the proposed intervention elements back to the original interview themes and quotations. For example, the call for “structured training in clinical leadership” was derived from sub-themes including “Continuous professional development and skill acquisition” and “Professional aspirations and anticipated roles”.
- Stakeholder-proposed components: Components proposed during the consensus meeting were documented with their source, indicating which participant or group proposed them. The small-group brainstorming generated initial proposals, and the plenary discussion refined and consolidated these proposals.
- Literature-derived components: The literature review presented at the meeting identified evidence-based elements from existing leadership development programs, which informed the structure and content of the intervention components.
- Legislatively informed components: Romanian legislation, Law no. 278/2015, and Government Emergency Ordinance no. 144/2008 informed the content of modules related to professional autonomy, scope of practice, and legal accountability.

It is important to distinguish between three primary sources of intervention elements, which carry different implications for the interpretation of the intervention’s origin:

- Participant-derived elements: Elements that were proposed or substantively modified by OTCNSs and other stakeholders during the consensus meeting. These include the specific duration of mentorship, extended from 3 to 6 months, the protected time allocation, refined to 1 h/week, the frequency of case forums, reduced from weekly to monthly, the quarterly reflective sessions, and the expansion from three to four curriculum modules.
- Literature- and researcher-derived elements: Elements that were initially proposed by the research team based on the targeted literature review and subsequently approved by participants. These include the overall four-component structure and the specific content of Curriculum Modules A, B, and C, which were derived from the literature and unanimously approved by participants without substantive modification.
- Legislation-derived elements: Elements informed by Romanian legislation, particularly Law no. 278/2015 and Government Emergency Ordinance no. 144/2008, which shape the content of modules related to professional autonomy, scope of practice, and legal accountability.

This distinction is important because it clarifies that the intervention was not entirely participant-generated. While the overall process was co-constructed, in the sense that stakeholders actively reviewed, modified, and approved all elements, the initial provenance of different components varied. The term “co-constructed” should therefore be understood as describing the collaborative refinement and validation process, not as implying that every component originated directly from participants. Several proposed components were discussed and modified during the consensus meeting:

- Mentorship duration: Initially proposed as three months of formal mentorship, this was extended to six months following discussion about the time needed for new OTCNSs to develop confidence and competence in the specialist role.
- Protected time: The proposal for “dedicated time for leadership activities” was refined to “one hour of protected time per week” to make it more specific and actionable.
- Case discussion frequency: Monthly interdisciplinary case forums were agreed upon, with the understanding that this would be reviewed after six months and adjusted if needed.
- Curriculum modules: The initial proposal for three modules was expanded to four modules following participant input on the importance of changing management skills.

The following proposed components were discussed but not included in the final intervention:

- Formal leadership rotation: A proposal for OTCNSs to rotate through leadership roles was rejected due to concerns about continuity of care and the need for stable mentorship relationships.
- External leadership program: A proposal to use an externally provided leadership training program was rejected in favor of a locally developed program tailored to the specific clinical context and legislative requirements.

The specific operational details, including six-month mentorship, one protected hour per week, monthly case forums, and quarterly reflective sessions, were derived from the consensus discussion. Participants based these recommendations on their experience with existing educational and mentorship activities, the typical learning trajectory for new OTCNSs, and the practical constraints of the clinical environment. These details represent a negotiated compromise between ideal design and realistic feasibility, as reflected in the discussion notes.

### 2.6. Quality Appraisal and Evaluative Criteria

To establish the methodological rigor and scientific integrity of this study, the evaluation criteria for qualitative inquiry outlined by Lincoln and Guba [46] were systematically implemented, encompassing credibility, transferability, dependability, and confirmability.

Credibility was secured through prolonged engagement with the clinical context and data source triangulation. The primary author embedded themself within the field setting to appreciate the operational realities of the specialized department. Furthermore, combining individual semi-structured interviews with multi-perspective expert consensus meetings provided data source triangulation, capturing an expansive, multi-layered view of clinical leadership dynamics.

Transferability was achieved by providing a highly descriptive, contextual narrative of the study setting—the Orthopedics and Traumatology Department of a major multi-specialty tertiary referral hospital—and the precise inclusion parameters of the Orthopedic and Trauma Clinical Nurse Specialists (OTCNSs). This transparent description allows future investigators to evaluate the relevance and applicability of our structural findings to equivalent healthcare infrastructures globally.

Dependability was maintained through a rigorous, well-documented operational pathway. All raw audio recordings were transcribed verbatim, and data analysis followed a clear, iterative thematic structure. To ensure coding consistency, collaborative multi-coder review sessions were organized among the research team, where the first and second authors together reviewed the coding and thematic structure across all transcripts. These sessions served to verify coding decisions and refine the thematic framework, minimizing subjective variance.

Confirmability was preserved by maintaining an explicit, transparent audit trail. This log detailed every phase of the research timeline, from early raw interview transcripts and initial coding frameworks to the final thematic syntheses verified during the collaborative consensus meetings. This open structural record ensures that the eventual conceptual model maps directly to the actual experiences of the participants rather than reflecting the personal assumptions of the research team.

### 2.7. Ethical Approvals and Participant Protections

The study was conducted in accordance with the Declaration of Helsinki [47]. Ethical approval was obtained from the Medical Ethics Committee for the Drug Study of the Targu Mures County Emergency Clinical Hospital [48]. All participants provided written informed consent prior to participation, with the consent form clearly explaining the purpose and nature of the study, what participation involves, the voluntary nature of participation and right to withdraw, how data will be collected, stored, and used, measures taken to ensure confidentiality and anonymity, and that anonymized responses and direct quotes may be used in publications. Data anonymization was ensured by creating an alphanumeric coding system, allowing only the lead researcher to know participant identities. All recordings and transcripts are stored in a password-protected secure folder, accessible exclusively to the research team.

## 3. Results

The results are presented in two main sections. The first section describes the findings from the semi-structured interviews conducted with Orthopedic and Trauma Clinical Nurse Specialists (OTCNSs), including participant characteristics, the thematic analysis, and a detailed narrative of the five identified macro-themes. The second section presents the outcomes of the consensus meeting, which co-constructed the constituent elements of a complex intervention aimed at developing clinical leadership within the orthopedic department.

### 3.1. Semi-Structured Interviews

#### 3.1.1. Cohort Characteristics and Participant Flow

Semi-structured interviews were conducted with seven OTCNSs representing the various clinical sub-specialties within the Orthopedics and Traumatology Department, including trauma, elective joint replacement, and outpatient fracture clinics. Of the 14 eligible OTCNSs in the department, seven were purposively selected and invited to participate. All seven provided informed consent and participated with a 100% acceptance rate. The majority were male (71.4%), employed full-time (90–100%), and had between 3 and 10 years of experience in the OTCNS role. The duration of the interviews ranged from 28 to 55 min (mean: 40 min).

The consensus meeting was attended by 11 participants: nine OTCNSs, including the seven interviewees plus two additional OTCNSs, one Head Nurse from the Orthopedics Department, and one Clinical Researcher with expertise in nursing leadership and implementation science. All 11 invited participants attended, with a 100% acceptance rate. It is important to note that the saturation achieved in the interview phase applies specifically to this narrowly defined departmental sample of seven OTCNSs and does not claim broader thematic exhaustiveness regarding clinical leadership in orthopedic nursing across different settings or contexts. The pilot interview participant did not attend the consensus meeting.

Table 1 details the characteristics of the interview participants and the consensus meeting attendees separately. The combined total of 18 participants reflects the sum of the interview sample (*n* = 7) and the consensus meeting sample (*n* = 11), with the seven overlapping OTCNSs counted in both phases to accurately report the composition of each phase. This combined total does not represent 18 unique individuals.

With respect to specialist qualifications, 4 of 7 interviewees (57.1%) held a DAS or Master’s degree, while the remaining 3 held specialist nurse status obtained through other pathways recognized by OAMGMAMR, including postgraduate specialist courses in orthopedics and traumatology nursing. All participants held active membership in OAMGMAMR and were employed in the functional OTCNS role in the department. The demographic characteristics reflect a predominantly male sample, which is consistent with the composition of the orthopedic nursing workforce in this specific setting. Most participants were employed full-time, 90–100%, had 6–15 years of total nursing experience, and had completed postgraduate qualifications in nursing. Experience as an OTCNS ranged from 1 to 10 years, with most participants having 3–6 years of experience in the specialist role.

#### 3.1.2. Qualitative Findings and Thematic Framework

From the hybrid deductive–inductive thematic analysis of the interview transcripts, 19 initial sub-themes were identified, which were subsequently grouped into 5 overarching themes that captured the essence of clinical leadership as perceived by orthopedic OTCNSs. These overarching themes are: (1) Individual attributes and professional capabilities, (2) Interprofessional collaboration and relational dynamics, (3) Team frameworks and organizational culture, (4) Role-based leadership and functional autonomy, and (5) Patient-centered trajectories. Table 2 provides a summary of the thematic network diagram, including the frequency of references across interviews.

#### 3.1.3. Narrative of Overarching Themes

Overarching theme, (1) Individual attributes and professional capabilities, explored the intrinsic qualities and professional skills that participants considered foundational to clinical leadership in the orthopedic setting. A dominant sub-theme was clinical decision-making and independent practice, described as the ability to make independent, evidence-informed decisions in complex and fast-paced clinical situations. Participants emphasized that orthopedic nurses frequently encounter patients with acute pain, mobility restrictions, and rapidly changing postoperative conditions, requiring swift, autonomous clinical judgement. As one participant stated: “In orthopedics, you often must decide quickly about pain management or mobilization protocols before the doctor arrives. You need the confidence to act, and that comes from knowing your specialty inside out” (OTCNS4); “I want to grow into a leadership role, but I don’t know what that path looks like in orthopedics. There’s no clear career progression that shows me how to get from where I am to where I want to be” (OTCNS2)”; “You can’t lead if you don’t know what you’re talking about. The junior nurses look to you when they don’t know how to do something, and if you’re not up to date, they won’t trust you” (OTCNS6); “You have to be able to handle the pressure. Orthopedics can be chaotic, multiple traumas coming in at once, patients in pain, families asking questions. If you fall apart, everyone falls apart” (OTCNS4).

The overarching theme (2) Interprofessional collaboration and relational dynamics, grouped together themes related to relational dynamics that either foster or impede clinical leadership. Horizontal peer collaboration and interdisciplinary dialogue was a prominent sub-theme, appearing six of the seven interviews. Participants stressed that orthopedic care is inherently multidisciplinary, involving surgeons, anesthetists, physiotherapists, occupational therapists, and social workers. Effective clinical leadership was perceived as the ability to navigate these complex professional boundaries, facilitate shared decision-making, and ensure that the nursing perspective was valued in care planning: “Clinical leadership here means being the voice of the patient in the multidisciplinary team meeting. You must collaborate with surgeons who are focused on the operation, and with physiotherapists who focus on movement. The nurse leads by holding the whole picture together” (OTCNS7); “Good communication isn’t just about talking. It’s about knowing who needs to know what, and when. If you don’t communicate clearly, things get missed, and patients suffer” (OTCNS5); “Things change all the time. New protocols, new equipment. You have to be willing to learn and adapt, and help others do the same. That’s part of leadership” (OTCNS1).

Engaging with clinical superiors and nursing administration, specifically the Head Nurse and Nurse Manager, was also pivotal. Participants described a dual dynamic: when superiors were supportive, empowering, and open to dialogue, OTCNSs felt enabled to exercise leadership. Conversely, a rigid hierarchical approach was perceived as a significant barrier. As one participant noted: “My manager trusts my decisions about patient allocation and complex wound care. That trust gives me the legitimacy to lead my junior colleagues. Without it, I would just be another nurse” (OTCNS3).

The overarching theme, (3) Team frameworks and organizational culture, explored how the broader organizational context and micro-culture of the orthopedic department influenced clinical leadership. The orthopedic clinical ecosystem and workplace climate was a central concern. Participants described a dual reality: on one hand, a strong sense of collegiality and mutual support among nursing staff; on the other, the pervasive strain of understaffing and high workloads. A positive climate was viewed as fertile ground for leadership to flourish: “When the team supports each other, it’s easy to step up and suggest improvements. But when everyone is stressed and overloaded, leadership feels like a burden rather than an opportunity” (OTCNS5); “The way the ward is organized makes a huge difference. If the workflow is inefficient, you’re constantly firefighting. Good leadership means thinking about how to make the system work better” (OTCNS3).

While most participants described a positive climate of collegiality, some also identified challenges. One participant noted: “When the team supports each other, it’s easy to step up and suggest improvements. But when everyone is stressed and overloaded, leadership feels like a burden rather than an opportunity’ (OTCNS5). Another participant offered a contrasting perspective: “I don’t think leadership is about being the loudest voice. Sometimes the quietest nurse, who just does her job well and helps others, is the real leader’ (OTCNS3). These divergent views highlight that clinical leadership can be enacted through different styles and in different contexts.

Continuous professional development and skill acquisition were highlighted as essential organizational factors. Participants expressed a strong desire for specific training in clinical leadership, communication, and conflict resolution, which they felt was currently lacking. Many noted that their clinical expertise in orthopedics had been developed through experience, but leadership skills had not been systematically cultivated: “We have excellent technical skills, but nobody taught us how to lead a team, how to give constructive feedback, or how to handle a difficult colleague. We need structured training, not just ad hoc learning” (OTCNS1).

In the overarching theme, (4) Role-based leadership and functional autonomy, participants explored the nuances of leadership specifically within their capacity as OTCNSs. Role clarity was considered foundational. Many participants felt that the expectations of the OTCNS role in orthopedics were not always clearly articulated, leading to confusion about their leadership responsibilities and authority: “I am a Specialist Clinical Nurse, but sometimes I feel like a supernumerary staff nurse. I need a clear definition of what leadership means in my job description, not just a list of clinical tasks” (OTCNS6).

A clear distinction emerged between formal leadership, associated with hierarchical positions such as Head Nurse, and informal leadership, associated with clinical expertise and peer credibility: “Our most experienced senior nurse doesn’t have a manager title, but everyone goes to her for advice. She influences how we practice orthopedic nursing, that’s real clinical leadership” (OTCNS2).

The final overarching theme, (5) Patient-centered trajectories, captured participants’ perceptions of the impact of clinical leadership on patient outcomes. Participants articulated their belief that effective clinical leadership enhances patient well-being and satisfaction. They described how, in their experience, when clinical leaders were actively engaged in coordinating care, patients appeared to experience better pain management, fewer complications, and smoother transitions from hospital to rehabilitation or home. As one participant stated: “Good clinical leadership in orthopedics means the patient gets their physiotherapy on time, their pain is controlled, and they understand their home care plan. That leads to better mobility and fewer readmissions” (OTCNS3); “I always ask my patients how they’re feeling, not just about their pain score. Are they comfortable? Can they move? Do they understand what’s happening? Those are the things that matter to them” (OTCNS7). These statements reflect participants’ perceptions based on their clinical experience, rather than objectively measured outcomes.

The theme of quality assurance and orthopedic safety protocols was also prominent. Participants described how leadership behaviors—such as proactive risk assessment and adherence to evidence-based protocols (for example, Venous Thromboembolism (VTE) prophylaxis, fall prevention, and timely escalation of clinical concerns)—were perceived to contribute to patient safety. Participants expressed the view that clinical leadership supports improved quality metrics in acute care settings. As one participant noted: “When a nurse leader is on the ward, you notice the difference. Protocols are followed more carefully, and patients seem to do better” (OTCNS2). These perceptions, while consistent with the literature, represent participant beliefs and hypotheses rather than demonstrated causal relationships.

#### 3.1.4. Current Leadership Development Landscape

To address the third research objective, examining the extent to which leadership development initiatives are currently integrated into routine daily practice, participants were asked to describe existing opportunities, their availability, and their utilization.

Participants identified several existing activities that, while not formally designated as leadership development, provide some opportunities for leadership skill acquisition, including informal mentorship, continuing professional development, and multidisciplinary team meetings.

Experienced senior nurses often provide ad hoc guidance to junior colleagues. However, this is unstructured and depends on individual willingness: “Our most experienced senior nurse doesn’t have a manager title, but everyone goes to her for advice” (OTCNS2). Despite this, participants noted that this informal support is inconsistent and not systematically available to all staff. The hospital provides some continuing professional development opportunities, primarily focused on clinical skills and technical competencies. However, these rarely address leadership competencies specifically: “We have excellent technical skills, but nobody taught us how to lead a team” (OTCNS1). Regular team meetings provide some opportunity for interprofessional collaboration and communication skill development, though these are focused on patient care rather than leadership development per se.

As regards availability, participants reported that formal leadership development programs are not available within the department or the hospital, structured mentorship programs are not available, although informal mentorship occurs inconsistently, and role-specific orientation for OTCNSs is not provided, despite recognition that the role differs from general nursing.

Participants described their engagement with available activities. All participants reported seeking informal mentorship from experienced colleagues when needed, though this was not systematic. Most participants (6/7) reported attending available continuing professional development activities, but noted that these focused on clinical rather than leadership skills. All participants participated in multidisciplinary team meetings, viewing these as opportunities to practice communication and collaboration skills.

### 3.2. Consensus Meeting Outcomes

Following the thematic analysis, a three-hour consensus meeting was convened in May 2026. The meeting was attended by nine OTCNSs representing various orthopedic sub-specialties and levels of experience, one Head Nurse from the Orthopedics Department, and one Clinical Researcher with expertise in nursing leadership and implementation science from the George Emil Palade University of Medicine, Pharmacy, Science, and Technology of Targu Mures. It is important to emphasize that the consensus reached represents stakeholder consensus within this local group, rather than evidence of general professional consensus. The results should be interpreted with this contextual limitation in mind.

The meeting commenced with a 20 min presentation of the interview findings, including the five overarching themes, the sub-themes, and illustrative quotations. This was followed by a 10 min review of relevant literature on clinical leadership development interventions and a brief overview of Romanian legislation concerning nursing autonomy including Law no. 278/2015 and Government Emergency Ordinance no. 144/2008.

Participants unanimously concurred that the identified themes accurately reflected the reality of the orthopedic department. Several participants provided additional examples supporting the themes, and no participant expressed disagreement with the thematic framework. As one participant stated: “This is exactly what we experience every day. It’s good to see it articulated so clearly” (OTCNS5).

The discussion then turned to identifying gaps in current leadership development. Participants identified the following critical gaps: Absence of a structured orientation pathway for new OTCNSs; Lack of formal leadership training for nurses at any career stage; Insufficient mentorship and coaching support in daily practice; Limited opportunities for peer learning and case-based reflection; Unclear role expectations and boundaries between OTCNSs and other nursing roles; Inadequate protected time for leadership or professional development activities.

Participants unanimously agreed, 100% agreement by show of hands, on the need for a structured, multicomponent intervention to address these gaps. No divergent views were expressed on this fundamental question. As one participant noted: “We’ve been asking for this for years. It’s time we had a proper system” (OTCNS2).

Participants then engaged in structured discussion to develop the intervention components. The process proceeded as follows:

- Small-group brainstorming (30 min): Participants were divided into three mixed groups (by experience and sub-specialty) and asked to brainstorm potential intervention components addressing the identified gaps. Each group generated 5–8 proposals.
- Plenary presentation (20 min): Each group presented its proposals, which were recorded on a flip chart by the facilitator.
- Discussion and refinement (20 min): The full group discussed each proposal, merging similar ideas, refining specifications, and identifying any missing elements. This discussion was characterized by active participation from all attendees, with the Head Nurse contributing primarily on issues of feasibility and resource availability, while OTCNSs focused on content and practical implementation.
- Prioritization and voting (15 min): Participants voted on each proposed component using a show of hands. The consensus threshold (≥80% agreement) was met for all components that were ultimately included.
- Finalization (10 min): The final list of components was reviewed and confirmed by the group.

All final intervention components received ≥85% agreement. The mentorship duration of six months received 91% agreement (10/11); one participant preferred a longer period. The protected time allocation, one hour per week, received 100% agreement (11/11). The curriculum modules were unanimously approved, 100% (11/11). The monthly and quarterly frequencies for peer circles received 91% agreement (10/11). While these levels of agreement demonstrate strong support for the proposed components within this local group, it is important to acknowledge that all voting was conducted publicly, with the Head Nurse present. Although anonymous voting was offered as an option, all participants chose public voting. This may have introduced an element of social desirability or hierarchical influence. The high levels of agreement should therefore be interpreted as stakeholder consensus within this specific local context, rather than as evidence of general professional consensus.

The following components were discussed and modified during the process:

- Mentorship duration: Initially proposed as three months, extended to six months following discussion. Rationale: “Three months isn’t enough for a new specialist to feel confident. They need at least six months of regular support” (OTCNS4).
- Protected time: Refined from “dedicated time” to “one hour per week” to make it specific and measurable.
- Peer circle frequency: Initially proposed as weekly, reduced to monthly. Rationale: “Weekly would be too much with our workload. Monthly is realistic and still valuable” (OTCNS7).

The following proposed components were discussed but not included:

- Formal leadership rotation: A proposal for OTCNSs to rotate through leadership roles was rejected. Initial vote: 6/9 (67%) in favor. After further discussion, a second vote was held: 6/9 (67%) in favor, remaining below the 80% threshold. Concerns raised included potential disruption to patient care and the need for stability in mentorship relationships.
- External leadership program: A proposal to use an externally provided training program was rejected. Initial vote: 8/11 (73%) in favor. After further discussion, a second vote was held: 8/11 (73%) in favor, remaining below the 80% threshold. Participants preferred a locally developed program tailored to their specific context.

Table 3 provides a comprehensive decision trail for the specific intervention elements, documenting the proposed source, alternatives considered, decision process, supporting evidence, and source category for each element.

**Figure 1:**
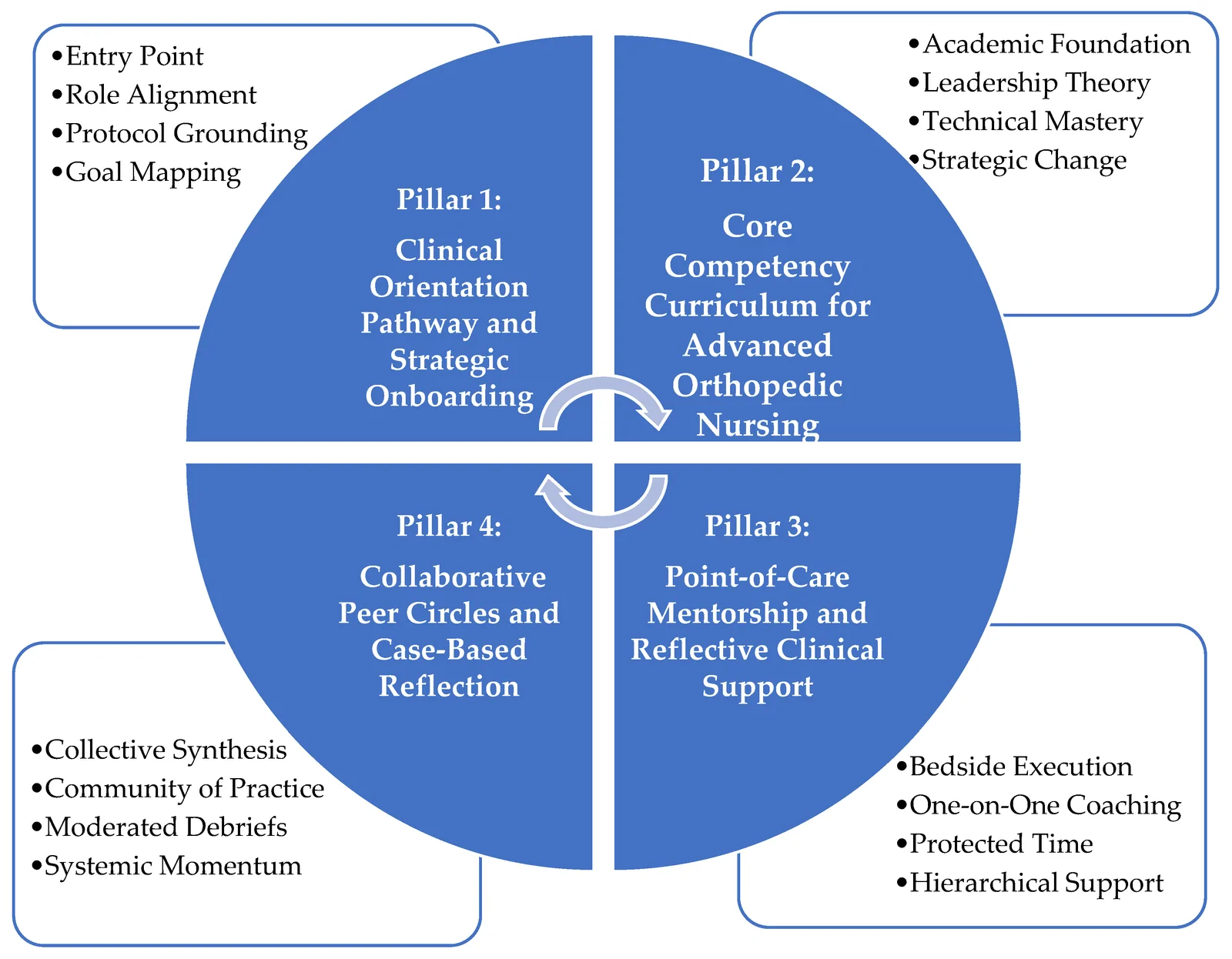
Proposed iterative cycle of OTCNS leadership development intervention components designed to operate iteratively to support the development of clinical leadership among OTCNS.

Based on these discussions, to systematically drive clinical leadership development within the department, a complex intervention was designed around four interconnected structural dimensions, which are intended to support broader organizational change and operational autonomy according to Law no. 278/2015: Component 1: Clinical Orientation Pathway and Strategic Onboarding, initiating baseline alignment regarding specialized role functions; Component 2: Core Competency Curriculum for Advanced Orthopedic Nursing delivering targeted technical knowledge on clinical protocols, for example VTE prophylaxis management; Component 3: Point-of-Care Mentorship and Reflective Clinical Support, providing real-time, on-the-job expert coaching to reinforce autonomous decision-making; Component 4: Collaborative Peer Circles and Case-Based Reflection, facilitating interactive, team-led debriefs to foster collective resilience and peer learning (Figure 1).

The four candidate components: (1) Clinical Orientation Pathway and Strategic Onboarding, (2) Core Competency Curriculum, (3) Point-of-Care Mentorship and Reflective Clinical Support, and (4) Collaborative Peer Circles and Case-Based Reflection, are designed to operate iteratively to support the development of clinical leadership among Orthopedic and Trauma Clinical Nurse Specialists. The cyclical nature reflects the understanding that leadership development is not a linear process but requires continuous reinforcement, practice, and reflection. Each component reinforces and builds upon the others: orientation provides foundational knowledge; curriculum develops competencies; mentorship applies learning in practice; and peer circles sustain reflection and collective learning. The cycle is intended to be repeated as OTCNSs progress through their careers and as new challenges emerge.

In the case of the first dimension, (1) Clinical Orientation Pathway and Strategic Onboarding, participants stressed the necessity of a formal induction and orientation pathway for OTCNSs entering the orthopedic department. This introductory trajectory should include: a structured welcome and role orientation session led by the Head Nurse and a senior OTCNS; a clear definition of the OTCNS role, responsibilities, and expected leadership behaviors; an introduction to the hospital’s quality and safety frameworks alongside specific evidence-based protocols relevant to orthopedic care, such as enhanced recovery after surgery protocols, VTE prevention, and pressure injury prevention; and collaborative goal-setting with a mentor covering both clinical and leadership development objectives.

As regards the second structural component, (2) Core Competency Curriculum for Advanced Orthopedic Nursing, a modular training programme was proposed, to be delivered in partnership with the local university and the hospital’s continuing education department. The mandatory modules should include: Module A—Foundations of Clinical Leadership, focusing on definitions, theoretical frameworks, for example transformational leadership, shared governance, and self-assessment of leadership styles; Module B—Effective Communication and Interprofessional Collaboration, covering active listening, non-violent communication, conflict resolution, and giving/receiving constructive feedback; Module C—Evidence-Based Orthopedic Practice and Quality Improvement, detailing critical appraisal, implementing guidelines, for example Specialty Commission of Ministry of Health recommendations, local protocols, and leading small-scale quality improvement projects; and Module D—Empowerment and Change Management, emphasizing organizational culture, leading change, and building resilient teams.

For the third operational component, (3) Point-of-Care Mentorship and Reflective Clinical Support, recognizing the gap between theoretical knowledge and practical application, participants strongly advocated for a supportive clinical environment. This experiential component includes: the assignment of a designated mentor for each OTCNS (initially a senior OTCNS or Head Nurse) for a minimum of six months; regular one-on-one coaching sessions to review clinical leadership experiences, discuss challenges, and reinforce bedside learning; and the provision of protected time, for example one hour per week, for OTCNSs to engage in leadership activities and reflective practice.

The final collaborative component, (4) Collaborative Peer Circles and Case-Based Reflection, emphasizes collective learning and peer support through: monthly interdisciplinary case discussion forums where OTCNSs can present complex orthopedic cases and discuss the leadership dimensions of coordinating care; quarterly reflective practice sessions facilitated by a trained moderator, allowing OTCNSs to share experiences, learn from each other, and build a community of practice; and the formal formation of a clinical leadership working group within the orthopedic department to sustain momentum and advocate for continued organizational support.

To guarantee structural alignment and methodological transparency, a conceptual matrix was constructed (Table 4).

Horizontal peer collaboration and interdisciplinary dialogue were prominent sub-themes across all interviews, with participants consistently emphasizing the importance of lateral communication in exercising clinical leadership. This prominence, evidenced by the depth and richness of participant accounts across all seven interviews, suggests that peer-to-peer collaboration is a central mechanism through which OTCNSs currently exercise clinical leadership.

This framework systematically maps the five emergent overarching themes and their respective orthopedic-specific sub-themes directly onto the study’s four foundational research objectives, ensuring a clear analytical pathway from raw qualitative data to the eventual complex intervention design.

Table 5 maps each final intervention component to its sources, including the interview theme, stakeholder input, the literature evidence, and legislative basis.

#### 3.2.1. Intervention Logic Model and Program Theory

Consistent with the MRC framework for complex interventions, the proposed intervention was conceptualized through a program theory specifying the relationships between inputs, activities, mechanisms, outputs, and outcomes. Table 6 presents the logic model.

The intervention is expected to operate through several interconnected mechanisms:

- Knowledge enhancement: The orientation and curriculum components provide OTCNSs with foundational knowledge about leadership, legal responsibilities, evidence-based practice, and change management;
- Skill development: Through didactic training, mentorship, and reflective practice, OTCNSs develop practical skills in communication, clinical decision-making, and interprofessional collaboration;
- Confidence building: Mentorship and peer support create a safe environment for OTCNSs to experiment with leadership behaviors and develop their professional identity as clinical leaders;
- Community formation: Peer circles and working groups establish a community of practice that sustains leadership development beyond the formal intervention period;
- Structural empowerment: The intervention aims to embed leadership development into routine practice through protected time, formal mentorship structures, and organizational recognition of the OTCNS role.

#### 3.2.2. Implementation Considerations and Key Uncertainties

Consistent with the MRC framework [37], the research team and consensus participants identified several implementation considerations, key uncertainties, and potential barriers.

Key uncertainties surrounding the intervention focus on its feasibility, acceptability, fidelity, mechanisms, and sustainability. First, feasibility concerns whether the proposed intervention components can be implemented with existing resources and staffing levels in the department. Acceptability relies on whether OTCNSs and other department staff will engage with the intervention components as intended. Additionally, fidelity remains uncertain regarding whether the intervention can be implemented consistently across different clinical sub-specialties and with different mentors. It is also unclear if the hypothesized mechanisms, specifically knowledge, skills, confidence, and community will operate as expected to produce the intended outcomes. Finally, sustainability poses a question of whether the intervention can be sustained over time without ongoing external support.

Resource and economic considerations for the intervention involve staff time, mentor training, curriculum development, and facilitation. First, staff time is a critical factor, as the intervention requires protected time for OTCNSs to participate in orientation, training, mentorship sessions, and peer circles, with a one-hour-per-week allocation identified as the minimum feasible amount given current workload pressures. This represents a direct cost to the department in terms of reduced clinical capacity during protected time. Second, mentor training will be necessary, as senior OTCNSs and the Head Nurse would require training in mentorship and coaching skills, demanding both time and potential external expertise. This represents an upfront investment that may yield returns through improved staff retention and reduced turnover costs. Third, curriculum development is required for the four modules, which involve creating or adapting training materials, potentially in partnership with the local university. This may involve costs for content development, expert consultation, and materials production. Finally, facilitation of peer circles and reflective sessions will require trained individuals, creating a need for external support or the specialized training of internal staff. Ongoing facilitation costs should be considered in the sustainability planning.

Implementation barriers for the project include challenges related to workload, hierarchical culture, staff turnover, resource constraints, and competing priorities. High patient volume and unpredictable admissions create a demanding workload that may limit the time available for intervention activities, despite protected time allocations. Additionally, a traditional medical hierarchical culture may resist expanded nursing leadership roles. Staff turnover also poses a risk, as sudden changes in staffing can disrupt mentorship relationships and continuity. Furthermore, resource constraints such as a limited budget for training and release time may negatively affect implementation. Finally, other organizational initiatives act as competing priorities that could vie for crucial attention and resources.

Areas requiring refinement center on tailoring to sub-specialties, flexible delivery, technology integration, and outcome measurement. First, the curriculum and mentorship may need tailoring to fit different clinical sub-specialties, such as trauma, elective surgery, and outpatient care. Second, flexible delivery is essential, meaning training and peer circle formats must adapt to accommodate shift work and unpredictable schedules. Third, technology integration should be considered, specifically looking at virtual or hybrid formats for training and peer circles to increase overall accessibility. Finally, outcome measurement needs development to ensure the project has appropriate measures for evaluating feasibility, acceptability, and preliminary effectiveness.

It is important to acknowledge that patients and the public were not involved in the development of this intervention prototype. This was a deliberate decision at this early stage, given the focus on understanding the professional perspectives and organizational context necessary for designing a credible intervention. However, patient and public involvement is essential for future development phases, particularly for refining the intervention to ensure it is patient-centered and acceptable to the populations served. The research team plans to involve patients and family caregivers in the subsequent feasibility and refinement phases to incorporate their perspectives on care coordination, communication, and patient-centered outcomes.

## 4. Discussion

The aim of this study was to investigate how Orthopedic and Trauma Clinical Nurse Specialists (OTCNSs) working within a specialized Orthopedics and Traumatology department perceive their leadership roles, and to identify the structural components required to build a sustainable leadership intervention. By anchoring our analysis within a generic qualitative approach, we captured a dense, multi-layered view of clinical leadership operating at the intersection of legislative intent and institutional reality. The emergent thematic framework demonstrates that while advanced nursing autonomy is formally recognized by national statutes, its day-to-day execution relies heavily on lateral, peer-to-peer relationships and ad hoc improvisations rather than formalized organizational scaffolding [50].

Horizontal peer collaboration and interdisciplinary dialogue were prominent across the interview data, with participants consistently emphasizing the importance of effective communication and shared decision-making in the multidisciplinary orthopedic team. This theme appeared in six of the seven interviews and was supported by rich descriptions of how collaboration functions in practice. However, the frequency of coding does not demonstrate that this was the “dominant” leadership mechanism; rather, the thematic interpretation was based on the depth, consistency, and explanatory power of participants’ accounts. This heavy reliance on horizontal care coordination reflects a broader European trend where patient safety [51], clinical excellence [52], and rapid postoperative mobilization [53] are increasingly dependent on interprofessional synergy [54] rather than top-down command structures.

A deeper structural friction point is suggested when examining the interplay between formal and informal leadership domains. Our data revealed that participants frequently discussed “Statutory mandates and structural accountability” (27 coded passages) and “Organic and situational leadership actions” (27 coded passages) with similar frequency. However, the qualitative content of these discussions differed substantively: when participants described statutory mandates, they often referenced formal role definitions and legal frameworks that theoretically support autonomous practice; when they discussed situational leadership actions, they described the informal, often reactive leadership behaviors they felt compelled to deploy in their daily work. This pattern, evident across multiple interviews, suggests a potential gap between the formal recognition of nursing autonomy in Romanian legislation [28] and the practical experience of leadership at the clinical unit level. As one participant noted: “The law says we have autonomy, but in practice, I still need to ask permission for things that should be within my scope” (OTCNS6). This discrepancy warrants further investigation.

This qualitative pattern, evident across multiple interviews, points to a potential theory-to-practice gap within the clinical setting [55]. European healthcare systems are placing an increasing emphasis on advanced competencies [56] and independent practice [57], principles that have already been incorporated into Romanian legislation via Government Emergency Ordinance (OUG) no. 144/2008, as amended by Law no. 278/2015 [28]. Yet, despite this legal foundation, participants’ accounts suggest that formal institutional pathways for enacting leadership remain underdeveloped. As one participant stated: “I am a Specialist Clinical Nurse, but sometimes I feel like a supernumerary staff nurse. I need a clear definition of what leadership means in my job description” (OTCNS6). This perception of a gap between legislative intent and daily practice was expressed by multiple participants.

The legislative framework for nursing practice in Romania provides several strong provisions that support the development of clinical leadership among specialist nurses by ensuring their professional autonomy and continuous growth. Under Article 6 (a) of OUG 144/2008, nurses have the legal right to independently assess healthcare needs, as well as plan, organize, and deliver nursing services, establishing a firm foundation for autonomous decision-making. Furthermore, Article 6 (h) of the same ordinance grants them the authority to evaluate the quality of care they provide to directly improve daily professional practices, while Article 11 (1) firmly establishes their right to practice in an independent capacity, bringing the exact level of accountability required for true leadership. Finally, this entire framework is reinforced by the Order of General Nurses, Midwives and Medical Assistants (OAMGMAMR), which actively mandates ongoing professional development and the mastery of advanced skills throughout a nurse’s career.

This heavy reliance on ad hoc problem-solving indicates that formal institutional pathways and organizational validation within the hospital hierarchy remain underdeveloped or constrained by deeply rooted traditional clinical structures. Consequently, moving leadership development beyond individual training toward genuine broader organizational change requires a deliberate transition. The four operational components of our proposed complex intervention framework provide a practical pathway to address this need, potentially bridging the gap between legislative intent and clinical practice.

This study’s findings have particular significance within the Romanian healthcare context. Romania implemented its first national nursing leadership programme in 2022 [49], designed to move leadership development beyond individual training toward broader organizational change. European healthcare systems place increasing emphasis on advanced competencies and independent nursing practice, principles already incorporated into Romanian legislation. The Order of General Nurses, Midwives and Medical Assistants has actively promoted professional autonomy, leadership, and innovation. However, the present study suggests that despite these national developments, a significant gap persists at the clinical unit level, with no structured pathway existing for developing clinical leadership among orthopedic OTCNSs. The intervention prototype co-constructed in this study represents a candidate approach to addressing this gap, pending feasibility testing and evaluation. The Romanian experience may offer transferable insights for designing context-sensitive training programmes in other transitioning health systems, although such transferability requires empirical investigation.

### 4.1. Comparative Analysis with the Baseline Framework

When contrasting these findings with the baseline study conducted by Palermo et al. [34], several critical socio-cultural and clinical divergences emerge, despite a shared adherence to the dual-phase Medical Research Council (MRC) architectural design. Both investigations arrived at a five-dimension macro-thematic structure and a four-component circular intervention loop. However, it is important to emphasize that this structural similarity may partly reflect the predetermined analytical scaffold: the interview guide and methodological architecture were explicitly adapted from the source study. Therefore, the convergence should not be interpreted as independent empirical confirmation of a universal five-domain model of clinical leadership. Rather, the similarity reflects the shared methodological framework, while the internal text densities and operational focuses reflect distinct institutional realities.

The primary divergence lies in the demographic configuration and acute positioning of the study populations. The cohort in this research was heavily male-dominated (71.4%), reflecting the specific gender layout of the orthopedic and trauma workforce within the Romanian emergency public system. Furthermore, the clinical setting, an exceptionally high-volume, tertiary trauma referral center in the Transylvania region, infused the sub-themes with a baseline of acute crisis management. Where the framework by Palermo et al. [34] evaluated specialist clinical nurse leadership across broad, multi-specialty environments, our findings are deeply anchored to immediate, high-stakes musculoskeletal interventions, such as complex polytrauma coordination and strict complication mitigation.

An analytical comparison reveals that the Romanian cohort may experience a tension between formalized authority and improvised leadership. In our matrix, “Organic and situational leadership actions” and “Statutory mandates and structural accountability” were discussed with similar frequency, 27 coded passages each. However, the qualitative content of these discussions differed substantively: when participants described statutory mandates, they often referenced formal role definitions and legal frameworks; when they discussed situational leadership actions, they described the informal, often reactive leadership behaviors they felt compelled to deploy in their daily work. This pattern, evident across multiple interviews, suggests a potential gap between the formal recognition of nursing autonomy in Romanian legislation [28] and the practical experience of leadership at the clinical unit level. As one participant noted: “The law says we have autonomy, but in practice, I still need to ask permission for things that should be within my scope” (OTCNS6). This discrepancy warrants further investigation.

While both studies successfully co-constructed a four-component framework, the operational delivery within our components reflects distinct local needs. Under Component 2 “Core Competency Curriculum”, our participants explicitly demanded university-partnered, academic modular programs, developed alongside local institutions like the George Emil Palade University of Medicine, Pharmacy, Science and Technology of Targu Mures to combat a historical lack of structured leadership training in the regional curriculum. Additionally, within Component 1 and Component 3, the clinical execution was tied directly to highly standardized, measurable orthopedic indicators, such as Venous Thromboembolism (VTE) chemical prophylaxis schedules and early mobilization tracking. This contrasts with the more generalized leadership development milestones proposed in the baseline model.

### 4.2. Originality and Added Value of the Present Study

The relationship between this study and the Palermo et al. framework warrants explicit discussion. The present study was designed as a contextual adaptation and application of an established methodological approach, rather than a fully independent or de novo inquiry. This design choice was intentional and reflects a commitment to building on existing knowledge while generating new, context-specific insights. A comparative analysis of the original and adapted versions is presented in Table 7.

The originality and added value of this study lie in several domains. First, the study applies the Palermo et al. methodological architecture to a distinctly different clinical setting, a high-volume, tertiary trauma center in Romania, as opposed to the multi-specialty general hospital setting of the original study. This application generates new knowledge about the specific challenges and opportunities for clinical leadership in orthopedic trauma nursing, a specialty characterized by high patient acuity, rapid clinical decision-making, and complex multidisciplinary coordination.

Second, the study demonstrates the transferability and contextual adaptability of the methodological approach to a different healthcare system, specifically a post-socialist European system undergoing rapid professionalization and legislative reform. The explicit integration of Romanian legislation, Law no. 278/2015, Government Emergency Ordinance no. 144/2008 [28] and professional regulatory frameworks represents a contextual innovation not present in the original study.

Third, the intervention prototype developed in this study is a preliminary, locally co-designed prototype tailored to the specific needs, resources, and constraints of the Romanian orthopedic trauma setting, but it has not yet been tested for feasibility or effectiveness. While the four-component structure mirrors that of the original study, reflecting the inherent logic of comprehensive leadership development: orientation, training, practice support, and peer learning, the specific operational details, curriculum content, and implementation strategies were co-constructed with Romanian stakeholders and reflect their articulated needs.

Fourth, by conducting this study in Romania, the authors extend the geographic and cultural reach of the clinical leadership literature, contributing to the growing body of knowledge about nursing leadership development in transitioning healthcare systems [49]. The Romanian experience may provide potentially relevant insights for other transitioning healthcare systems, although transferability requires empirical evaluation in other contexts seeking to develop clinical leadership among specialist nurses.

In summary, this study should be understood as a contextual adaptation that systematically builds upon an existing methodological approach, rather than a replication or validation study. The thematic similarity to the original study at the macro-level, comprising five themes and four intervention components, reflects the shared methodological architecture. It does not constitute independent empirical validation of a universal five-domain model of clinical leadership. The specific content, operational details, and contextual integration represent genuinely new empirical contributions that are grounded in the Romanian data.

This study examined OTCNSs’ perceptions of their role and clinical leadership within a single orthopedic trauma department in Romania. Through qualitative interviews and a consensus meeting, the study identified perceived leadership development needs and generated candidate intervention components tailored to the local context.

This study did not measure actual leadership behaviors or competencies; assess patient outcomes (for example, pain management, complications, readmissions); evaluate organizational performance or quality metrics; test the feasibility, acceptability, or effectiveness of the proposed intervention; examine the fidelity of intervention implementation; assess the resource requirements or cost-effectiveness of the intervention; or investigate unintended consequences of the intervention.

### 4.3. Limitations of the Study

While this study provides important insights into developing clinical leadership, several limitations must be acknowledged. First, the investigation was conducted within a single center, the Orthopedics and Traumatology Department of the Targu Mures County Emergency Clinical Hospital. Consequently, the findings regarding institutional workflows and cultural climates may not be fully generalizable to other public or private medical institutions across Romania or wider regional frameworks. Second, the sample size, consisting of seven individual semi-structured interviews and eleven subsequent consensus meeting participants, was small. While data saturation was achieved within this narrowly defined departmental sample, this cohort reflects a specific group of specialized professionals, which may limit the breadth of perspectives captured. The saturation claim should therefore be understood as contextual, applying to this specific department and workforce, rather than as evidence of broader thematic exhaustiveness regarding clinical leadership in orthopedic nursing generally. Third, the sample was predominantly male, 71.4% of the interview cohort. Although this accurately reflects the unique composition of the orthopedic nursing workforce in this regional trauma center, it limits the transferability of these findings to broader, traditionally female-dominated nursing populations. Finally, while participants’ experiential accounts suggest a strong link between active nursing leadership and improved clinical delivery, this qualitative study did not directly measure patient-centered outcomes or track objective health indicators.

An additional limitation relates to the methodological relationship with the Palermo et al. study [34]. The present study was designed as a contextual adaptation of this existing approach, not as a fully independent inquiry. While this design choice was intentional and enabled systematic comparison across contexts, it also means that some aspects of the findings, particularly the macro-thematic structure and the four-component intervention architecture, are likely influenced by the source framework. The authors have attempted to distinguish between elements that were predetermined, including methodological structure, interview dimensions, consensus procedure, and those that emerged from the Romanian data as regards specific sub-theme content, operational details, and legislative integration. However, complete independence from the source framework cannot be claimed, and readers should interpret the findings with this in mind. Importantly, the structural similarity between the two studies should not be interpreted as independent empirical confirmation of a universal five-domain model of clinical leadership, as the analytical scaffold was explicitly adapted from the source study.

The risk of deductive identification is an important consideration in this study. The Orthopedics and Traumatology Department is a named single-center setting with a small specialist workforce, 14 OTCNSs total. The demographic characteristics reported in Table 1, including gender distribution, age ranges, and years of experience could, in combination with the departmental context, potentially allow identification of individual participants by those familiar with the department. To mitigate this risk, several measures were implemented: (1) an alphanumeric coding system was used to anonymize participant identities; (2) quotations are attributed only to broad participant codes, for example OTCNS1–OTCNS7, without additional identifying details; (3) potentially identifying details within quotations were reviewed and, where necessary, generalized while preserving meaning; and (4) the research team carefully reviewed all quotations to ensure they did not inadvertently reveal participants’ identities. Despite these measures, readers should be aware that complete anonymity cannot be guaranteed in a small, specialized workforce, and the findings are presented with this limitation in mind.

The consensus meeting procedure warrants consideration of potential group conformity and social desirability effects. The first author’s dual role as researcher and meeting facilitator, combined with the presence of hierarchical relationships among participants, including the Head Nurse, may have influenced participant responses. Several measures were implemented to mitigate these effects, as detailed in Section 2.4.2: participants were informed that all perspectives were equally valued; the Head Nurse was asked to allow junior participants to speak first; small-group brainstorming was conducted in mixed groups; the facilitator actively encouraged contributions from quieter participants; and anonymous voting was offered as an option. However, despite these measures, complete elimination of social desirability effects cannot be guaranteed. Notably, all participants chose public voting, and the Head Nurse was present throughout the meeting. The reported 100% agreement on the need for an intervention and the ≥85% agreement on all final components should therefore be interpreted with awareness of the potential for group conformity, particularly given that participants with different levels of organizational authority were present. The research team attempted to document any divergent views, as evidenced by the rejected proposals for formal leadership rotation (67% agreement) and external leadership programs (73% agreement), which demonstrate that participants were willing to express disagreement when they had it. However, the absence of more substantial disagreement may reflect, in part, the hierarchical dynamics of the clinical setting. Consequently, the results should be described as stakeholder consensus within this local group, rather than evidence of general professional consensus. This limitation should remain prominent when interpreting the findings and the proposed intervention components.

In summary, the consensus reached in this study represents the collective judgment of a specific group of stakeholders within a single department at a single point in time. While the process was structured and transparent, the results should not be interpreted as evidence of broader professional consensus regarding clinical leadership development in orthopedic nursing.

### 4.4. Future Research Directions

In close alignment with the progressive steps mandated by the Medical Research Council (MRC) framework, future research must transition from the development phase to evaluation and feasibility testing. Immediate next steps should concentrate on conducting localized pilot studies to evaluate the acceptability, practical feasibility, and implementation fidelity of the co-constructed four-component intervention cycle. Subsequent multi-center evaluation studies are required to expand the generalizability of this framework across diverse clinical specialties and hospital tiers within transitioning healthcare systems.

Importantly, future quantitative and mixed-methods research should integrate clear, validated clinical and organizational performance metrics to measure actual systemic impact. These outcome indicators should encompass clinical safety metrics, including incident reporting frequencies, patient-reported outcome measures (PROMs), and strict adherence rates to evidence-based protocols, such as Venous Thromboembolism (VTE) prophylaxis schedules and early mobilization timelines; organizational outcomes, including longitudinal tracking of specialist nurse turnover rates, clinical burnout scores, and measurable shifts in team culture using validated leadership self-assessment tools; and relational transformations, including multi-source evaluation mapping of changes in interprofessional collaboration and communication efficacy across traditionally rigid medical hierarchies.

Ultimately, these future research directions will serve to translate qualitative insights into empirical, data-driven validation, supporting the sustained integration of advanced nursing practice globally.

## 5. Conclusions

This qualitative study examined perceptions of clinical leadership among Orthopedic and Trauma Clinical Nurse Specialists at a major Romanian trauma center. The findings revealed that clinical leadership is perceived as shaped by a dynamic interplay of personal, relational, institutional, and patient-centered variables, and that significant gaps exist in formal leadership development opportunities at the clinical unit level. While the five-theme structure aligns with the source framework, this alignment reflects the adapted methodological scaffold rather than independent empirical validation of a universal model.

The study generated a preliminary, contextually tailored intervention prototype comprising four candidate components: clinical orientation and onboarding, competency-based curriculum, point-of-care mentorship, and collaborative peer circles. These components were co-constructed through a consensus process involving frontline OTCNSs, nursing leadership, and a clinical researcher within this local group, and were informed by the thematic analysis findings, relevant literature, and Romanian legislative requirements. The consensus reached represents stakeholder consensus within this specific departmental context, rather than evidence of general professional consensus, and should be interpreted with this limitation in mind.

The proposed intervention is a locally developed prototype that has not yet been tested for feasibility, acceptability, or effectiveness. The findings reported in this study reflect participant perceptions and experiences, not objectively measured leadership behaviors or patient outcomes. Claims about the intervention’s capacity to produce “systemic transformation” or serve as a “policy blueprint” are premature.

Future research should prioritize: (1) feasibility testing of the proposed intervention components in the clinical setting; (2) assessment of acceptability among OTCNSs and other stakeholders; (3) refinement of the intervention based on feasibility findings; (4) development of appropriate outcome measures; and (5) evaluation of the intervention’s effectiveness through robust study designs, including consideration of organizational, patient, and implementation outcomes. Patient and public involvement should be incorporated into subsequent development phases to ensure the intervention is responsive to patient priorities and values.

The Romanian experience may offer transferable insights for other transitioning health systems seeking to develop clinical leadership among specialist nurses, although the transferability of the findings requires empirical investigation across different contexts.

## Figures and Tables

**Table 1:** Cohort characteristics by study phase, presenting demographic and professional characteristics of participants in the interview phase and the consensus meeting phase.

Cohort Characteristics	Interviews (*n* = 7) *n* (%)	Consensus Meeting (*n* = 11) *n* (%)	Total Reported Across Phases ^1^ (N = 18) *n* (%)
Sex			
Male	5 (71.4)	6 (54.5)	11 (61.1)
Female	2 (28.6)	5 (45.5)	7 (38.9)
Age, years			
20–35	3 (42.9)	4 (36.4)	7 (38.9)
36–50	3 (42.9)	5 (45.5)	8 (44.4)
>50	1 (14.3)	2 (18.2)	3 (16.7)
Postgraduate Qualification (DAS/Master’s ^2^)			
Yes	4 (57.1)	7 (63.6)	11 (61.1)
No	3 (42.9)	4 (36.4)	7 (38.9)
Total Nursing Experience, years			
2–5	1 (14.3)	2 (18.2)	3 (16.7)
6–15	5 (71.4)	5 (45.5)	10 (55.6)
16–25	1 (14.3)	4 (36.4)	5 (27.8)
Employment Status (%)			
70–80%	1 (14.3)	1 (9.1)	2 (11.1)
90–100%	6 (85.7)	10 (90.9)	16 (88.9)
Experience as OTCNS, years			
1–2	3 (42.9)	4 (36.4)	7 (38.9)
3–6	3 (42.9)	5 (45.5)	8 (44.4)
7–10	1 (14.3)	2 (18.2)	3 (16.7)

^1^ The total of 18 reported participants reflects the sum of the seven interview participants and the 11 consensus meeting participants. The seven OTCNSs who participated in the interviews were a subset of the nine OTCNSs who attended the consensus meeting. Therefore, this combined total does not represent 18 unique individuals. The consensus meeting also included one Head Nurse and one Clinical Researcher who were not part of the interview phase. The combined total is presented to accurately reflect the participation across both phases of the study. Therefore, for every characteristic, the consensus meeting value is equal to or greater than the interview value. ^2^ Postgraduate Qualification (DAS/Master’s) refers specifically to the Diploma of Advanced Studies (DAS) or Master’s degree in nursing. Participants who did not hold a DAS/Master’s may nevertheless have held specialist nurse status obtained through other pathways recognized by OAMGMAMR, such as postgraduate specialist courses or the professional competency examination.

**Table 2:** Thematic network diagram showing the 19 sub-themes grouped into 5 overarching themes identified from thematic analysis of the seven interviews.

Overarching Theme	Sub-Theme	*n* Sources ^1^	Total Citations ^2^
1. Individual attributes and professional capabilities	Clinical decision-making and independent practice	7	25
	Professional aspirations and anticipated roles	7	22
	Evidence-based expertise and technical mastery	6	18
	Mindful leadership styles	7	28
	Intrinsic attributes and psychological resilience	7	19
2. Interprofessional collaboration and relational dynamics	Engaging with clinical superiors and nursing administration	7	26
	Horizontal peer collaboration and interdisciplinary dialogue	6	18
	Clinical communication pathways and information exchange	5	15
	Driving clinical innovation and adaptability	6	22
3. Team frameworks and organizational culture	The orthopedic clinical ecosystem and workplace climate	6	20
	Continuous professional development and skill acquisition	6	28
	Operational frameworks and workflow design	7	18
	Structural empowerment and professional agency	6	28
	Professional validation and merit appreciation	7	25
4. Role-based leadership and functional autonomy	Professional role configuration and boundary definition	7	24
	Statutory mandates and structural accountability	7	27
	Organic and situational leadership actions	7	27
5. Patient-centered trajectories	Patient-reported quality of life and musculoskeletal well-being	6	15
	Structural quality assurance and orthopedic safety protocols	6	18

^1^ *n* Sources: The number of interview transcripts (out of 7) in which the sub-theme was identified. A source count of 7 indicates the sub-theme appeared in all interviews. ^2^ Total Citations: The total number of text segments or quotations coded under this sub-theme across all transcripts. These counts are presented descriptively to indicate the breadth of coverage of each sub-theme, but do not constitute evidence of relative importance. Frequency of coding is influenced by multiple factors including interview duration, questioning, and coding decisions, and should not be interpreted as a measure of conceptual significance.

**Table 3:** Decision trail for specific intervention elements, documenting the proposed source, alternatives considered, decision-making process, supporting evidence, and source category for each of the co-constructed intervention components.

Specific Element	Proposed by	Alternatives Considered	Decision Process	Evidence/Quotation	Source
Mentorship duration (6 months)	Consensus discussion (modified from initial proposal)	3 months; 12 months	Extended from 3 to 6 months following discussion. Vote: 91% agreement.	“Three months isn’t enough for a new specialist to feel confident. They need at least six months of regular support” (OTCNS4)	Participant-derived (modified from initial researcher proposal)
Protected time (1 h/week)	Consensus discussion (refined from “dedicated time”)	“Dedicated time” (unspecified); 30 min/week; 2 h/week	Refined to specific measurable unit. Vote: 100% agreement.	Based on participants’ practical feasibility assessment	Participant-derived (refined from researcher proposal)
Monthly case forums	Consensus discussion (reduced from weekly)	Weekly; bi-weekly; monthly	Reduced from weekly to monthly based on workload concerns. Vote: 91% agreement.	“Weekly would be too much with our workload. Monthly is realistic and still valuable” (OTCNS7)	Participant-derived (modified from initial proposal)
Quarterly reflective sessions	Consensus meeting proposal	Monthly; bi-monthly; quarterly	Proposed by participants and accepted.	Proposed by OTCNSs based on experience with reflective practice	Participant-derived
Curriculum Module A: Foundations of Clinical Leadership	Research team (informed by literature)	No alternatives proposed	Unanimously approved (100%).	“Nobody taught us how to lead a team” (OTCNS1); the literature [41,42]	Literature-derived with participant validation
Curriculum Module B: Communication and Collaboration	Research team (informed by literature)	No alternatives proposed	Unanimously approved (100%).	Participants identified communication as essential; the literature [3,7]	Literature-derived with participant validation
Curriculum Module C: Evidence-Based Orthopedic Practice	Research team (informed by literature and interviews)	No alternatives proposed	Unanimously approved (100%).	“We have excellent technical skills but need structured training” (OTCNS1); the literature [22,23]	Interview- and literature-derived
Curriculum Module D: Empowerment and Change Management	Consensus discussion (expanded from initial 3 modules)	Initial proposal for 3 modules	Expanded to 4 modules following participant input. Unanimously approved (100%).	Participants requested skills for leading change; legislation supports autonomy [28]	Participant-derived (expansion)

**Table 4:** Conceptual matrix mapping research objectives to the five emergent qualitative themes, their corresponding sub-themes, and analytical focus.

Research Objective	Overarching Theme	Corresponding Sub-Themes	Analytical Focus/ Data Application
Objective 1: Investigate OTCNS Perceptions of Roles and Leadership Scope	Theme 1: Individual Attributes and Professional Capabilities Theme 4: Role-Based Leadership and Functional Autonomy	• Clinical decision-making and independent practice • Professional aspirations and anticipated roles • Professional role configuration and boundary definition • Statutory mandates and structural accountability	Evaluates how specialist nurses understand their legal boundaries, clinical duties, and professional identity under national frameworks.
Objective 2: Identify Elements and Behaviors Fostering Leadership Growth	Theme 1: Individual Attributes and Professional Capabilities Theme 2: Interprofessional Collaboration and Relational Dynamics	• Evidence-based expertise and technical mastery • Mindful leadership styles • Intrinsic attributes and psychological resilience • Horizontal peer collaboration and interdisciplinary dialogue	Pinpoints the exact psychological traits, technical clinical knowledge, and peer support systems that drive leadership growth at the ward level.
Objective 3: Examine Presence or Absence of Daily Leadership Interventions	Theme 3: Team Frameworks and Organizational Culture Theme 4: Role-Based Leadership and Functional Autonomy	• The orthopedic clinical ecosystem and workplace climate • Continuous professional development and skill acquisition • Operational frameworks and workflow design • Structural empowerment and professional agency • Professional validation and merit appreciation • Organic and situational leadership actions	Conducts a structural audit of current hospital operations, identifying systemic barriers like a lack of protected time and rigid medical hierarchies.
Objective 4: Identify Components for a Complex Intervention (MRC Framework)	Theme 2: Interprofessional Collaboration and Relational Dynamics Theme 3: Team Frameworks and Organizational Culture Theme 5: Patient-Centered Trajectories	• Engaging with clinical superiors and nursing administration • Clinical communication pathways and information exchange • Driving clinical innovation and adaptability • Patient-reported quality of life and musculoskeletal well-being • Structural quality assurance and orthopedic safety protocols	Feeds data directly into the co-construction of the 4 intervention components to improve clinical safety protocols, such as VTE prophylaxis.

**Table 5:** Intervention component traceability matrix linking each of the four co-constructed intervention components to its source(s).

Intervention Component	Source(s)	Provenance	Evidence/Justification
Component 1: Clinical Orientation Pathway and Strategic Onboarding			
- Structured welcome and role orientation	Interview Theme 4: Role-based leadership (sub-theme: Professional role configuration)	Participant-derived	“I need a clear definition of what leadership means in my job description” (OTCNS6); Consensus discussion
- Clear definition of OTCNS role and responsibilities	Interview Theme 4: Statutory mandates; Legislation (Law 278/2015)	Legislation-derived	Participants identified role ambiguity as a key barrier; legal framework mandates professional autonomy
- Introduction to quality and safety frameworks	Interview Theme 5: Structural quality assurance	Literature-/researcher-derived	“We need to know what the protocols are and how to apply them” (OTCNS1); the literature on patient safety [18,19]
- Collaborative goal-setting with mentor	Consensus meeting proposal	Participant-derived	Proposed by OTCNSs, supported by mentorship literature [32]
Component 2: Core Competency Curriculum			
- Module A: Foundations of Clinical Leadership	Interview Theme 1: Mindful leadership styles; Theme 3: CPD and skill acquisition	Literature-/researcher-derived	“Nobody taught us how to lead a team” (OTCNS1); the literature on leadership development [41,42]
- Module B: Communication and Collaboration	Interview Theme 2: Horizontal peer collaboration; Clinical communication pathways	Literature-/researcher-derived	Participants identified communication as essential for leadership; the literature on interprofessional collaboration [3,7]
- Module C: Evidence-Based Orthopedic Practice	Interview Theme 1: Evidence-based expertise; Theme 5: Quality assurance	Literature-/researcher-derived	“We have excellent technical skills but need structured training” (OTCNS1); evidence-based practice literature [22,23]
- Module D: Empowerment and Change Management	Interview Theme 3: Structural empowerment; Consensus discussion	Participant-derived (expansion)	Participants requested skills for leading change; legislation supports professional autonomy [28]
Component 3: Point-of-Care Mentorship			
- Designated mentor for six months	Consensus discussion (modified from 3 months)	Participant-derived	Supported by mentorship literature [32]; consensus vote: 91% agreement
- One-on-one coaching sessions	Consensus meeting proposal	Participant-derived	Proposed by OTCNSs to bridge theory-practice gap; the literature on reflective practice [11]
- Protected time (1 h/week)	Consensus discussion (refined from “dedicated time”)	Participant-derived	Consensus vote: 100% agreement; based on participants’ practical feasibility assessment
Component 4: Collaborative Peer Circles			
- Monthly interdisciplinary case forums	Consensus discussion (reduced from weekly)	Participant-derived	Consensus vote: 91% agreement; supported by literature on interprofessional learning [20,21]
- Quarterly reflective practice sessions	Consensus meeting proposal	Participant-derived	Proposed by OTCNSs; the literature on reflective practice and peer learning [6]
- Clinical leadership working group	Consensus discussion	Participant-derived	Proposed to sustain momentum and advocacy; the literature on organizational sustainability [49]

**Table 6:** Logic Model for the OTCNS leadership development intervention specifying the relationships between inputs, activities, mechanisms, outputs, and outcomes across short-term (6–12 months), medium-term (1–2 years), and long-term (2–5 years) timeframes.

Inputs	Activities	Mechanisms	Outputs	Outcomes
Resources:	Orientation & Onboarding:	Enhanced knowledge	Immediate:	Short-term (6–12 months):
- Dedicated mentors	- Structured welcome session	- Understanding of role	- Completion of orientation	- Increased role clarity
- Protected time allocation	- Role definition and responsibilities	- Legal and accountability	- Completion of 4 curriculum	- Enhanced clinical competence
- Training materials	- Protocol introduction	awareness	modules	- Improved communication skills
- University partnership	- Goal-setting with mentor	- Protocol familiarity	- Regular mentorship meetings	- Increased confidence
- Continuing education support				
	Competency Curriculum:	Enhanced skills		
Contextual factors:	- Four modules (leadership, communication, EBP, change management)	- Communication	Intermediate:	Medium-term (1–2 years):
- Legislative framework (Law 278/2015)		- Clinical decision-making	- Formation of peer circles	- Sustained mentorship relationships
- Organizational culture		- Leadership behaviors	- Working group established	- Peer learning community
- Workload and staffing		- Change management		- Improved interprofessional collaboration
- Existing CPD structures	Mentorship & Support:			
	- Designated mentor (6 months)	Enhanced confidence	Long-term:	Long-term (2–5 years):
Assumptions:	- Regular coaching sessions	- Self-efficacy	- Replication of mentorship	- Sustained leadership behaviors
- Participants willing to engage	- Protected reflection time	- Professional identity	across department	- Systemic organizational change
- Organizational support		- Leadership identity	- Integration into routine	- Enhanced patient outcomes ^1^
- Adequate resources	Peer Learning:		practice	- Improved staff retention
- Leadership valued	- Monthly case forums	Peer support		
	- Quarterly reflective sessions	- Collective learning		
	- Working group formation	- Community of practice		

^1^ Patient outcome are hypothesized based on participant perceptions and literature but were not measured in this study.

**Table 7:** Comparison of original and adapted methodological elements between the Palermo et al. [34] baseline study and the present Romanian adaptation.

Element	Palermo et al. (Original)	Present Study (Romanian Adaptation)	Modification Made
Setting	Multi-specialty general hospital, Italy	Tertiary trauma center, Romania	Changed clinical context
Participants (Interviews)	7 specialist clinical nurses	7 OTCNSs	Same number, different specialty
Consensus Meeting	11 participants (9 nurses, 1 manager, 1 researcher)	11 participants (9 OTCNSs, 1 Head Nurse, 1 researcher)	Same composition, different roles
Interview Dimensions	7 key dimensions	7 key dimensions (same structure)	Retained structure, rephrased for Romanian context
Overarching Themes	5 themes (similar structure)	5 themes (similar macro-structure)	Same macro-structure; different sub-themes
Distinctive Sub-themes	Not present	“Statutory mandates and structural accountability”, “The orthopedic clinical ecosystem”	New sub-themes emerged from Romanian data
Intervention Components	4 components (orientation, training, coaching, group work)	4 components (orientation, curriculum, mentorship, peer circles)	Similar structure; different operational details
Operational Details	Not specified in same detail	6-month mentorship, 1 h/week protected time, monthly forums	New details from Romanian consensus
Legislative Integration	Not present	Law 278/2015, OUG 144/2008	New: specific to Romanian context
Curriculum Modules	General content	4 specific modules (Leadership, Communication, EBP, Change Management)	Tailored to Romanian needs
Setting	Multi-specialty general hospital, Italy	Tertiary trauma center, Romania	Changed clinical context
Participants (Interviews)	7 specialist clinical nurses	7 OTCNSs	Same number, different specialty

## Data Availability

The datasets generated and/or analyzed during the current study are not publicly available due to the confidentiality of qualitative data and the small, identifiable participant sample, which would make deductive identification possible. De-identified transcripts, the codebook, analytical memos, and consensus meeting materials may be available from the corresponding author upon reasonable request, subject to institutional ethics committee approval and participant confidentiality considerations. The COREQ checklist is provided as Supplementary Material Table S1, the example of coding progression from raw data to theme is provided as Supplementary Material Table S2, and the three-hour meeting agenda is provided as Supplementary Material Agenda S1.

## Supplementary Materials

The following supporting information can be downloaded at https://www.mdpi.com/article/10.3390/healthcare14183020/s1, Table S1: COREQ Checklist; Table S2: Example of coding progression from raw data to theme; Agenda S1: The three-hour meeting.

## Abbreviations

The following abbreviations are used in this manuscript:

DAS	Diploma of Advances Studies
MRC	Medical Research Council
OAMGMAMR	The Romanian Order of General Nurses, Midwives and Medical Assistants
OTCNS	Orthopedic and Trauma Clinical Nurse Specialist
OUG	Government Emergency Ordinance
PROM	Patient-Reported Outcome Measure
VTE	Venous Thromboembolism

## Appendix A

Interview schedule

Section 1: Understanding of Clinical Leadership

1. What does the term “clinical leadership” mean to you in the context of your work in orthopedics?

- Prompt: What specific behaviors or actions come to mind when you think of a clinical leader in orthopedics? How would you describe a clinical leader to a new nurse joining your department?

2. In your experience, what distinguishes clinical leadership from managerial or administrative leadership in an orthopedic setting?

- Prompt: Can you give me an example where you have observed a difference between a manager and a clinical leader? Do you think a person can be both?

Section 2: Identification of Clinical Leaders in Orthopedics

3. When you think about your department, the Orthopedics and Traumatology Department, whom do you consider to be the people who demonstrate leadership behaviors?

- Prompt: What specific behaviors do they exhibit? Can you describe a situation where you observed someone demonstrating clinical leadership?

4. Among the people you identified, are there individuals who hold formal management positions, and are there others who do not? What is the difference in how they lead?

- Prompt: Do you think a person needs a formal title to be a clinical leader? Can you think of an example of an informal leader in your team?

5. If we shift the focus specifically to clinical practice at the bedside or in the ward, do you think clinical leadership exists here?

- Prompt: What does clinical leadership look like in the context of daily orthopedic patient care? Can you give me a practical example from your recent experience?

Section 3: Factors Fostering and Hindering Clinical Leadership

6. In your opinion, what factors within your work context, the Orthopedics and Traumatology Department and the broader hospital, foster or facilitate the development of clinical leadership?

- Prompt: What aspects of the team environment, organizational culture, or available resources support leadership development? Can you tell me about any significant episodes where you felt leadership was encouraged?

7. Similarly, what factors hinder or create barriers to the development of clinical leadership in your setting?

- Prompt: Are there organizational structures, workload issues, or interpersonal dynamics that make it difficult to exercise clinical leadership? Can you describe a situation where you felt your leadership potential was constrained?

8. If you could give a suggestion to hospital management or your department leadership to foster clinical leadership, what would you recommend?

- Prompt: What changes would make the biggest difference? What support would you need to develop your leadership skills?

Section 4: Personal Reflection on Role and Clinical Leadership

9. Thinking specifically about your role as an Orthopedic and Trauma Clinical Nurse Specialist, how do you perceive clinical leadership in relation to your own position?

- Prompt: Do you see yourself as a clinical leader? Why or why not? What aspects of your role allow you to demonstrate leadership?

10. In your usual work activities, what specific behaviors or activities do you consider to be expressions of clinical leadership?

- Prompt: Think about a typical day. What actions do you take that influence patient care, team practice, or clinical outcomes? Why do you consider these to be leadership behaviors?

11. What would you need, in terms of training, support, resources, or organizational changes, to further develop your clinical leadership?

- Prompt: Are there specific skills you would like to develop? What would help you feel more confident or effective as a clinical leader?

Section 5: Interprofessional Collaboration and Team Dynamics

12. Thinking about your role as an Orthopedic and Trauma Clinical Nurse Specialist and your activities within the interprofessional orthopedic team, which includes surgeons, physiotherapists, occupational therapists, and others, is it possible for you to demonstrate or enact clinical leadership?

- Prompt: Can you give me an example of a time when you exercised leadership within the multidisciplinary team? How did the team respond?

13. Continuing to think about your team and the professionals with whom you collaborate, are there specific relationships that foster the development of clinical leadership? And conversely, are there relationships that hinder it?

- Prompt: How does your relationship with the Head Nurse influence your leadership? How about your relationships with medical staff or allied health professionals? Can you describe a positive or challenging relationship dynamic?

14. How important is communication to your ability to lead effectively within the orthopedic team?

- Prompt: Can you give an example of how good communication supported your leadership? What about a time when poor communication created a barrier?

Section 6: Impact on Patient Care

15. Now, I would like you to shift your focus to your patients. What impact do you think clinical leadership has on the quality of care that orthopedic patients receive?

- Prompt: How does clinical leadership influence patient safety, patient satisfaction, or clinical outcomes such as pain management, mobility, or rehabilitation progress? Can you think of a specific example?

16. Do you believe that strong clinical leadership contributes to better coordination of care for orthopedic patients, especially those with complex trauma or multiple comorbidities?

- Prompt: What does comprehensive or holistic care look like in orthopedics? How does leadership help achieve this?

Section 7: Personal Satisfaction and Professional Development

17. Finally, I would like to return to you as a professional. Are there repercussions for your job satisfaction or emotions related to your clinical leadership style?

- Prompt: Do you feel satisfied with your current leadership capacity, or would you like to develop it differently? How does exercising leadership affect your sense of professional fulfilment, stress levels, or engagement?

18. Looking ahead, what are your aspirations for your professional development as a clinical leader in orthopedics?

- Prompts: Where do you see yourself in five years? What would help you get there?

19. Is there anything else you would like to add about clinical leadership in orthopedics that we haven’t discussed?
