# Supplementary material for: Developing Clinical Leadership in Orthopedic Nursing: A Qualitative Study to Identify the Components of a Complex Intervention
Source: Healthcare (Basel). 2026 Sep 15;14(18):3020. doi: 10.3390/healthcare14183020 (PMC13606198; doi:10.3390/healthcare14183020)
Supplement: Supplementary file 1 [file healthcare-14-03020-s001.zip › healthcare-4540204-supplementary.pdf]

SUPPLEMENTARY MATERIALS

Developing Clinical Leadership in Orthopedic Nursing: A Qualitative Study to Identify the Components of a Complex Intervention

Flaviu Moldovan[1,*] and Liviu Moldovan[2]

1 Orthopedics—Traumatology Department, Faculty of Medicine, George Emil Palade University of Medicine, Pharmacy, Science, and Technology of Targu Mures, 540142 Targu Mures, Romania

2 Faculty of Engineering and Information Technology, George Emil Palade University of Medicine, Pharmacy, Science, and Technology of Targu Mures, 540142 Targu Mures, Romania; liviu.moldovan@umfst.ro

* Correspondence: flaviu.moldovan@umfst.ro; Tel.: +40 754 671 886

Table S1. COREQ Checklist
Consolidated Criteria for Reporting Qualitative Studies (COREQ): 32-item Checklist

No.	Item	Guide Questions/Description	Reported on Page #
**Domain 1: Research team and reflexivity**			
Personal characteristics			
1	Interviewer /facilitator	Which author/s conducted the interview or focus group?	Page 6, Paragraph 1 (Section 2.1.2) "All semi-structured interviews were conducted by the first author..."
2	Credentials	What were the researcher's credentials? E.g. PhD, MD	Page 6, Paragraph 1 (Section 2.1.2) "a male researcher with a PhD in Medicine and MSc in Quality Management systems"
3	Occupation	What was their occupation at the time of the study?	Page 6, Paragraph 1 (Section 2.1.2) "employed as a university lecturer at the Faculty of Medicine and held an affiliate position within the Orthopedics and Traumatology Department"
4	Gender	Was the researcher male or female?	Page 6, Paragraph 1 (Section 2.1.2) "a male researcher"
5	Experience and training	What experience or training did the researcher have?	Page 6, Paragraph 1 (Section 2.1.2) "completed formal training in qualitative research methods, including a university-certified course in qualitative data analysis and thematic analysis with duration of 40 hours, and had previously conducted and published qualitative research in healthcare

			settings"
Relationship with participants			
6	Relationship established	Was a relationship established prior to study commencement?	Page 6, Paragraph 1 (Section 2.1.2) "the first author had established professional relationships with some members of the Orthopedics and Traumatology Department through prior collaborative research projects"
7	Participant knowledge of the interviewer	What did the participants know about the researcher?	Page 6, Paragraph 3 (Section 2.1.2) "participants were aware of the first author's affiliation with the department and his role as a researcher"
8	Interviewer characteristics	What characteristics were reported about the interviewer/facilitator?	Page 6, Paragraphs 2-4; Page 4, Paragraph 1 (Section 2.1.2) "held no hierarchical authority... measures implemented: participants informed voluntary, interviews in private setting, confidentiality assured, open-ended questions, consensus meeting facilitated with attention to all participants"
**Domain 2: Study design**			
Theoretical framework			
9	Methodological orientation and Theory	What methodological orientation was stated to underpin the study?	Page 4, Paragraphs 1-3 (Section 2.1) "generic qualitative design... prospectively designed as a contextual adaptation and empirical application of the methodological approach developed by Palermo et al. [34]"
Participant selection			
10	Sampling	How were participants selected?	Page 8, Paragraphs 2-3 (Section 2.3) "purposeful sampling according to reasoned choice... Seven OTCNSs were purposively selected from the 14 eligible OTCNSs to achieve this diversity"
11	Method of approach	How were participants approached?	Page 9, Paragraph 4 (Section 2.3) "Participants were contacted individually by the first author"
12	Sample size	How many participants were in the study?	Page 17, Paragraph 1; Table 1 (Section 3.1.1) "seven OTCNSs... 11 participants: nine OTCNSs... one Head Nurse... one Clinical Researcher"
13	Non-participation	How many people refused to participate or dropped out? Reasons?	Page 9, Paragraphs 2-4 (Section 2.3) "All seven invited OTCNSs agreed to participate... No refusals occurred... All 11 invited consensus meeting participants agreed to participate. No refusals occurred."
Setting			
14	Setting of	Where was the data	Page 9, Paragraph 1 (Section 2.4) "private

	data collection	collected?	office within the Orthopedics and Traumatology Department”
15	Presence of non-participants	Was anyone else present besides the participants and researchers?	Page 9, Paragraph 1 (Section 2.4) “No one other than the participant and the interviewer was present during any interview”
16	Description of sample	What are the important characteristics of the sample?	Page 17, Table 1 (Section 3.1.1) “Male 71.4%, Female 28.6%, Age, Post-graduate qualifications, Total Nursing Experience, Employment Status, Experience as OTCNS”
Data collection			
17	Interview guide	Were questions, prompts, guides provided by the authors? Was it pilot tested?	Page 9, Paragraph 3; Appendix A (Section 2.4) “The interview schedule comprised 19 open-ended questions organized across seven key dimensions... The interview guide was pilot-tested with one OTCNS... The full interview guide is provided in Appendix A”
18	Repeat interviews	Were repeat interviews carried out? If yes, how many?	Page 10, Paragraph 6 (Section 2.4) “No repeat interviews were conducted, as data saturation was achieved with the initial set of seven interviews”
19	Audio/visual recording	Did the research use audio or visual recording to collect the data?	Page 10, Paragraph 5 (Section 2.4) “Interviews were audio-recorded using a digital recorder with the participant's permission”
20	Field notes	Were field notes made during and/or after the interview or focus group?	Page 10, Paragraph 6 (Section 2.4) “Field notes were made immediately after each interview, documenting the interviewer's initial impressions, the setting, any contextual factors that may have influenced the interaction, and methodological observations”
21	Duration	What was the duration of the interviews or focus group?	Page 14, Paragraph 1; Page 6, Paragraph 6 (Section 3.1.1 and Section 2.4) “duration of interviews ranged from 28 to 55 minutes (mean: 40 minutes)”
22	Data saturation	Was data saturation discussed?	Page 11, Paragraph 1; Page 10, Paragraph 8 (Section 2.4.1 and Section 2.4) “Saturation was prospectively defined as the point at which three consecutive interviews yielded no new code or thematic content... Saturation was assessed after the fourth interview... after the fifth... definitively after the sixth when no new codes emerged... confirmed with the seventh interview”
23	Transcripts returned	Were transcripts returned to participants for comment and/or correction?	Page 10, Paragraph 1 (Section 2.4) “Preliminary findings, including the initial thematic framework and a summary of key codes and sub-themes, were presented to a subgroup of three interview participants for

			member checking... Full transcripts were not returned to participants to maintain the focus on thematic interpretation rather than individual-level verification”
**Domain 3: analysis and findings**			
Data analysis			
24	Number of data coders	How many data coders coded the data?	Page 13, Paragraphs 3-6 (Section 2.5) “The first author conducted the initial coding of all seven transcripts... the second author independently reviewed a random sample of three transcripts... conducted two 'multi-coder review sessions' where the first and second authors together reviewed the coding and thematic structure across all transcripts”
25	Description of the coding tree	Did authors provide a description of the coding tree?	Page 16, Table 2 (Section 3.1.2) “Thematic network diagram showing the 19 sub-themes grouped into 5 overarching themes”
26	Derivation of themes	Were themes identified in advance or derived from the data?	Page 18, Paragraph 2 (Section 2.5) “The analysis employed a hybrid approach combining deductive and inductive elements”
27	Software	What software, if applicable, was used to manage the data?	Page 10, Paragraph 3 (Section 2.5) “ATLAS.ti 23 software [45]”
28	Participant checking	Did participants provide feedback on the findings?	Page 10, Paragraph 1 (Section 2.4) “All three confirmed that the themes accurately reflected their experiences and perspectives. No substantive corrections were requested”
Reporting			
29	Quotations presented	Were participant quotations presented to illustrate the themes/findings?	Pages 19-21 (Section 3.1.3) “OTCNS4... OTCNS2... OTCNS6... OTCNS7... OTCNS5... OTCNS1... OTCNS3... OTCNS6... OTCNS2... OTCNS3... OTCNS2”
30	Data and findings consistent	Was there consistency between the data presented and the findings?	Throughout Section 3.1.3 and Section 3.2 - quotations support the thematic narrative
31	Clarity of major themes	Were major themes clearly presented in the findings?	Pages 19-21 (Section 3.1.3) “Overarching theme (1) Individual attributes and professional capabilities... Overarching theme (2) Interprofessional collaboration... Overarching theme (3) Team frameworks... Overarching theme (4) Role-based leadership... final overarching theme (5) Patient-centered trajectories”
32	Clarity of minor	Was there a description of diverse cases or	Page 20, Paragraph 4 (Section 3.1.3) “While most participants described a positive

	themes	discussion of minor themes?	climate of collegiality, some also identified challenges... Another participant offered a contrasting perspective... These divergent views highlight that clinical leadership can be enacted through different styles and in different contexts"

Table S2. Example of coding progression from raw data to theme

Raw Data Extract (Participant)	Initial Code	Sub-theme	Overarching Theme
"In orthopedics, you often must decide quickly about pain management or mobilization protocols before the doctor arrives. You need the confidence to act, and that comes from knowing your specialty inside out" (OTCNS4)	Quick decision-making; confidence from knowledge; specialty expertise	Clinical decision-making and independent practice	Individual attributes and professional capabilities
"Clinical leadership here means being the voice of the patient in the multidisciplinary team meeting. You must collaborate with surgeons who are focused on the operation, and with physiotherapists who focus on movement. The nurse leads by holding the whole picture together" (OTCNS7)	Voice of patient; multidisciplinary collaboration; holistic view	Horizontal peer collaboration and interdisciplinary dialogue	Interprofessional collaboration and relational dynamics
"I am a Specialist Clinical Nurse, but sometimes I feel like a supernumerary staff nurse. I need a clear definition of what leadership means in my job description" (OTCNS6)	Role confusion; unclear expectations; need for definition	Professional role configuration and boundary definition	Role-based leadership and functional autonomy

Agenda S1 - The three-hour meeting

The three-hour meeting followed this structured agenda:

1. Welcome and introduction (10 minutes)
2. Presentation of interview findings (20 minutes)
3. Brief review of literature and legislative context (10 minutes)
4. Open discussion of findings and identification of gaps (30 minutes)
5. Small-group brainstorming on intervention components (30 minutes)
6. Plenary presentation and discussion of proposed components (30 minutes)
7. Prioritization and refinement of components (20 minutes)
8. Development of implementation considerations (15 minutes)
9. Summary and next steps (15 minutes)
